# Perception of patient-centred care and its relationship with management outcomes and complications among patients with DM in Malawi

**DOI:** 10.1136/bmjopen-2024-090308

**Published:** 2025-07-05

**Authors:** Martha Thokozani Makwero, Adamson S Muula, Felix Chima Anyanwu, Innocent Maposa, Jude Igumbor

**Affiliations:** 1 Department of Family Medicine, Kamuzu University of Health Sciences, School of Medicine and Oral Health, Blantyre, Malawi; 2 School of Public Health, University of the Witwatersrand Faculty of Health Sciences, Johannesburg, Gauteng, South Africa; 3 Department of Public Health, Kamuzu University of Health Sciences, School of Global and Public Health, Blantyre, Malawi; 4 School of Public Health, University of the Witwatersrand, Faculty of Health Sciences, Johannesburg, Gauteng, South Africa; 5 Department of Biostastics, Stellenbosch University, Tygerberg, Capetown, South Africa

**Keywords:** Health Services, Chronic Disease, General diabetes, Health Education

## Abstract

**Abstract:**

**Background:**

Patient-centred care (PCC) is associated with better experiences for chronic care encounters and better outcomes. However, its assessment and resultant management outcomes have not been well documented in Malawi. As Malawi strives to institutionalise PCC in its quality-of-care initiatives, documenting its correlates and outcomes is a good starting point in the implementation and advocacy of PCC among chronic care patients, particularly those living with diabetes mellitus (DM).

**Aim:**

We sought to assess the level of diabetic patients’ perception of PCC and its relationship to self-efficacy, adherence and glycaemic control among patients with DM.

**Study setting:**

This study was done in DM clinics of two district and two central hospitals in southern Malawi.

**Research design and methods:**

This was a cross-sectional analytical study. We studied 607 subsequent consenting adult patients with DM. We assessed the level of perception of using a locally generated and validated tool and its correlation with self-efficacy, adherence and long-term glycaemic control after a medical encounter. We used K-means clustering, linear and logistic regression, and path analysis in the analysis.

**Outcomes:**

The study’s outcome measures included adherence, self-efficacy, long-term glycaemic control. Adherence included aspects related to medication, diet, lifestyle and appointment keeping and was assessed using the Hill-Bone tool. Self-efficacy was assessed using a Stanford self-efficacy tool while long-term glycaemic control was determined through haemoglobin A1c (HbA1c) point-of-care testing.

**Results:**

Overall, the mean score for PCC was 62.86 (SD 14.78). The study highlighted two groups of patients with statistically distinct mean scores of 51.6 (7.8) vs 77.1 (7.2) out of a possible 92 (p<0.001), respectively. More patients (n=234 out of 436), 55.7% belonged to the cluster with an average score of 51.56, highlighting that more patients perceived less PCC and that low perception was more prominent in the patient individualisation and involvement subscale. Path analysis showed that female sex and the presence of complications had lower PCC scores than males and those without complications. We found a non-significant relationship between PCC and self-efficacy. Having tertiary education was associated with a 6.4 increase in efficacy scores (β=6.36; 95% CI 1.96 to 10.76, p<0.05). Both PCC and self-efficacy were positive predictors of adherence independently. Having perceived better PCC was associated with a marginal 0.03-point increase in one’s adherence scores (β=0.03; 95% CI 0.01 to 0.04, p<0.001). The effect of efficacy on adherence was of similar magnitude. Self-efficacy and adherence were both positive predictors of blood sugar control independently. Better self-efficacy was associated with a 0.03 unit decrease in the level of HbA1c (β=−0.03; 95% CI −0.04 to −0.022, p<0.001). Additionally, as adherence scores went higher, there was a 0.15 unit decrease in HbA1c (β=−0.15; 95% CI −0.25 to −0.02, p<0.05) signalling better glycaemic control.

**Conclusions:**

Although this study did not find a direct correlation between PCC and glycaemic control, it has demonstrated that PCC medical encounters could potentially improve glycaemic control by enhancing patients’ adherence to their diabetes management plans. Even though PCC is not an end in itself, medical encounters that prioritise good relational ambience, patient involvement and capacitation are promising interventions in DM care, especially for patients with or at risk of DM complications. The general lack of patient individualisation and involvement elements found in the medical encounters in our study could explain why PCC hardly has an impact on self-efficacy. The study highlights the importance of PCC in DM patient management and outlines important innovative adaptations towards transforming medical education to equip and appraise interpersonal skills that have an impact on patient-reported experiences and outcomes.

**STRENGTHS AND LIMITATIONS OF THIS STUDY**

- The use of multiple methodological and analytical approaches in elucidating patient-centred care (PCC), the development of a measurement framework and the assessment of PCC and its correlates improves the validity of the findings.
- The use of multiple perspectives of patients, healthcare providers and policy-makers improves triangulation in the definition and reduces consumerism.
- Being a non-interventional study, it falls short of describing causal relationships between PCC and outcomes.
- PCC being a sociobehavioural phenomenon, it has other determinants that explain the relationships between itself and outcomes but were not considered in this study, such as social support and health literacy.
- There is the potential bias of the self-reported data on complications of diabetes mellitus due to social desirability, where participants might have been giving responses they thought would make the interviewers happy to gain favours in care.

## Introduction

 Patient-centred care (PCC) in medical encounters is associated with better experiences of care and outcomes, yet the extent to which patients perceive PCC and its associated outcomes has not been well documented in healthcare reform literature.^1^ Literature, which is largely from high-resourced contexts, represents documentation concerning objective assessment of PCC and causal studies that highlight its value in routine care.^2^,^7^ Some of these studies have highlighted the causal links and how PCC can impact patient-reported experiences and mediate outcomes such as adherence to care medication, lifestyle, appointment keeping and glycaemic control in the chronic care context.^4 6 8^ Adherence to care plans as an outcome of PCC is pivotal to the attainment of treatment goals among patients with diabetes mellitus (DM).^8 9^ While these studies may be of high quality, there is a scarcity of studies on such kinds of objective assessment and causal links between PCC and management outcomes in low-income and middle-income countries such as Malawi, particularly among chronic care patients, where such care approaches could make a difference in patient management.^1 10 11^

Perception refers to the way in which a construct is regarded, understood or interpreted and, ultimately, experienced by the respective actors.^12^ Individual actors’ sociodemographic characteristics, culture, prior experiences and disease conditions have the potential to shape perceptions about PCC.^13^ While the debate regarding whose perceptions between healthcare providers (HCPs) or users matter in the assessment of PCC is ongoing, studies have shown that measuring patients’ perceptions of PCC is a reliable gauge of the delivery of care and its responsiveness.^14^ Evidence suggests that patients’ perceptions are more predictive of outcomes than are providers’ perceptions and interaction observation.^213^,^15^ Therefore, in any context, the heavy inclination on patients’ perceptions in assessing what matters in PCC and to what extent it is being delivered is justifiable since they are beneficiaries and the central object of care.

As an approach to care, PCC entails that patients are active participants in their care and that healthcare systems and their respective teams respond to patient needs and preferences. PCC is underpinned by a functional provider–patient interaction during medical encounters.^16^ In the Malawi context, PCC is conceptually understood as an expected care process that incorporates warm patient reception, where the HCP consciously aims to reduce the patient-HCP power gap to harness a good long-term relationship. This creates a conducive atmosphere that allows the gathering of information that holistically identifies the individual’s specific problems and all possible interacting factors, ensuring timely access to care and medication.^17^ Such interaction thrives within different organisational layers, namely individual provider–patient interaction, institutional and broader system, and contextual levels.^18^,^20^

PCC complements the biomedical aspects of care by facilitating the attainment of better patient-reported experiences and health status outcomes.^7 11 21^ Documented evidence on PCC outcomes is not consistent because of poor conceptualisation of PCC and challenges with randomisation of the studies. However, the results on the same are still largely promising.^3^ Some outcomes include patient satisfaction, perceived quality of care, patient adherence to management plans, reduced emergency visits, complications, patient empowerment and self-efficacy.^3 7 22^ Attainment of such outcomes is affected by patient factors, HCP and contextual factors. Likewise, pathways linking PCC to quality outcomes may not be direct but through intermediary outcomes or factors.^23^

Amidst the growing complexity of disease management plans and rising patient’ expectations, care delivery needs to shift from the traditional acute, episodic and biomedical models to embrace more PCC approaches. In the latter, provider–patient encounters are capable of integrating both technical and interpersonal aspects of care and dealing with biopsychosocial patient demands over time with the provider as a resource.^24^ Furthermore, emphasis should be placed on the fact that such interactions should foster partnerships with patients, leveraging their capabilities towards individual and population health promotion.^24^ ^(p.6)^ This being the case, PCC interactions would be fundamental to quality management of chronic patients, especially in limited resource contexts such as Malawi’s, particularly in this era of growing chronic non-communicable diseases (NCDs).^25^ Thus, interactional elements of medical encounters ought to be given due attention, assessing their value in impacting outcomes explored.

While Malawi, in its quality management directorate (QMD) for the health sector and the NCD and injury policies, strives to improve the delivery of quality care through people engagement and consideration of patients’ perspectives in care design and delivery. Current efforts in this area are, however, found wanting.^25^ Actually, documentation of perceptions about the quality of received DM care is sparse.^1 21 26^ The current QMD efforts have focused on suggestion boxes, promotion of patient charters and patients’ satisfaction surveys, which do not effectively elicit patients’ perceptions towards care design and delivery, thereby falling short of allowing safe spaces for patients to be effective participants in care.^27 28^ It is, therefore, imperative to explore patients’ perceptions of PCC in the chronic care context and its associated factors in order to adequately inform the implementation of the set policies. We, therefore, set out to assess the extent of patients’ perceptions of PCC and its correlates, using patients with DM as a case study.

### Objective

To assess the extent to which patients with DM in Malawi perceive PCC during their medical encounters and its relationship to self-efficacy, patient adherence to management care plans and glycaemic control.

## Methods

### Study design and setting

This was a quantitative observational analytical study. We collected data from DM clinics in two central hospitals (Queen Elizabeth and Zomba) and two district hospitals (Chikwawa and Mulanje).

### Study population and sampling

Our study population consisted of patients with DM seeking care at DM clinics. We employed simple, convenient sampling to achieve high generalisability and representativeness to the population under study.^29^ Being an observational study that was comparing two independent means (PCC clusters), the sample size for the study was based on the assumption that the effect size (ES) for the difference in adherence (the primary outcome) between two clusters was 0.16 among diabetics.^30^ The study assumed an alpha value of 5% and a minimum power of 80%. To offset some sample dependence that may exist in the clinics, we scaled the sample size by a design effect of 1.5.^31^ Accounting for a 10% non-response rate, the final sample size was 703. However, we only managed to get 607 due to the COVID-19 pandemic as DM clinics were running half full. Less than a 10% response rate was registered.

All subsequent patients, who accessed DM care from providers between September and December 2021, were approached to participate. Those who were less than 18 years old, those too unwell to respond, and those who patronised the clinic for less than 6 months were excluded from the study. Data were collected using interviewer-administered questionnaires. All patients were required to give informed consent by signing a written consent form. All personal identifiers were removed as a step to ensure privacy and confidentiality in the study.

### Patient and public involvement

Patients were involved in identifying the important elements in PCC, and that information was included in the PCC tool to ensure that this study measures what matters to patients.

### Data collection tools and variable scoring

#### Patient centred care

PCC was collected using a locally generated and validated tool that is attached as online supplemental appendix 1. To improve cultural context relevance, this tool was developed de novo and validated from the same population as an earlier part of this study using a mixed-methods approach. The tool had 23 Likert-type, time-bound 5-point Likert scale; The scores were as follows: 1=never, 2=occasionally, 3=often, 4=all the time and 5 was assigned to a null answer such as ‘no comment’ or ‘I don’t know’. The tool had three subscales, namely relational ambience (10 items), individualisation and shared decision-making (7 items) and organisational subscale (6 items). Through a psychometric analysis, the tool exhibited acceptable reliability and validity in psychometric properties, with a Cronbach’s alpha of 0.94 (unpublished manuscript).

#### Self-efficacy

Self-efficacy was measured using the Stanford self-efficacy for diabetes tool attached as online supplemental appendix 2. This eight-item tool had high internal consistency with Cronbach alpha scores of 0.828 (n=189 DM patients). The tool was validated in different settings and has portrayed acceptable validity measures.^32 33^ The tool has been adapted for use in Malawi with patients with DM in a similar population, and it was shown to be suitable.^34^ It has a 10-point numerical scale and the rating used was 1–10, depicting the least and most confidence in patients’ ability to perform certain tasks related to DM self-care, including dealing with hypoglycaemic states.^35^

#### Adherence to management plans

Adherence was measured using an adapted 10-item Hill-Bone compliance tool that uses a time bound 4-point Likert scale,^36^ ranging from 1 ‘none of the times’ to 4, ‘all of the time’ as attached in online supplemental appendix 3. Although the tool was developed for hypertension, it was adapted for DM care because the two conditions share similar care components, namely, adherence to medication, lifestyle modification and appointment-keeping. The tool is a valid measure of adherence in different settings.^37 38^ It was preferred because it was validated in a comparable cultural setting; a black South African (SA) population with a Cronbach alpha score of 0.79.^36^ To conform to the rest of the variables in the study, the scoring in the Hill-Bone adherence scoring was reversed. Thus, 1 was the least desirable response, and 4 was the best score in PCC and adherence, with 1 representing ‘never’ and 4 representing ‘always’.

The tool was used as a total scale without disaggregation of its components (medication, diet, lifestyle and appointment keeping)

#### Long-term glycaemic control

Long-term glycaemic control was measured using the point-of-care haemoglobin A1c (HbA1c) test. The rapid test has good acceptability, is easy to use and is stable in Malawi’s temperatures. The test was used in a comparable situation in the country.^39^

#### Diabetic comorbidities and complications

Reported comorbidities and diabetic complications were assessed as a proxy of DM control.^40^ Participants were asked to report through recall if they had experienced any of the DM complications in the last 6 months, and the response was a ‘yes’ or ‘no’. We considered the following DM complications: eye problems, neuropathy, kidney problems, DM limb ulcers and amputations, hypoglycaemia, hyperglycaemia, heart problems, loss of consciousness and erectile problems.^40^

### Quantitative data management and analysis

#### General data management

Initial data exploration was done in STATA V.17 to ensure quality, completeness and to transform some variables, create and assign labels. We explored the data elements for normality, appreciated the patterns and assumptions and then applied the required constraints. Duplicates were removed while missing data and ‘not applicable’, ‘no comment’ and/or ‘don’t know’ responses, which were less than 5% of the total dataset, were assigned a mode because they occurred at random and do not really affect the variance of string object data.^41^ Extreme outliers were identified using box plots, and seven entries were removed, representing 1.2% of the total dataset. The data normality checks helped to ascertain the most suitable inferential statistics to be used.

#### Sociodemographic and disease characteristics

Descriptive analysis was done on sociodemographic and disease characteristics and PCC scores. Summarisation was done using means, medians and SD or medians for continuous data, while frequencies and proportions were used for categorical data and presented in tables. Inferential statistics of associations between endogenous and exogenous variables such as socioeconomic variables, comorbidities, complications, self-efficacy, adherence scores and reported DM complications were performed. The results were expressed as p values fixed at <0.05 and CIs.^42^ For descriptive analysis, the presence of complications and comorbidities was analysed as a binary variable (present or absent). Linear and logistic regressions, as well as structural equation models, were further used to assess relationships.

#### PCC scoring and categorisation

We used unsupervised K-means cluster analysis to identify the number and properties of the PCC clusters among our study population using R-Studio V.4.2.3. The clustering allowed us to probabilistically discriminate patients into low and high PCC based on responses to 23 items in the PCC scale. The patient classification into either high or low PCC was then used to determine the effect on self-efficacy and health outcomes, vis-à-vis adherence and glycaemic control. The R output was transferred back into STATA and, in turn, used to determine if the means between the two clusters were statistically distinct using an independent t-test. The PCC scores were, therefore, reported as both categorical variables (frequencies and percentage frequencies were used) and as continuous variables (means, SD, minimum-maximum and Interquartle Range were used) for the initial exploratory analysis. The clusters were labelled high and low depending on the mean values identified through K-means cluster analysis. This categorisation highlighted the extent to which PCC delivered might be adequate apart from determining the level at which it makes statistical differences in outcomes, a dimension that is essential in mediating outcomes clinically.^4 8^

The scores were aggregated on a global scale, subscales and per individual. The possible minimum and maximum PCC scores were 23 and 92, respectively, for an individual.

### Self-efficacy scoring

For self-efficacy, 1 meant the least confident, while 10 denoted the most confident. Self-efficacy scores per individual ranged from 8 to 80 and were analysed as a continuous variable in order not to lose detail and were summarised as aggregates using means, SD, median and IQR.

### Adherence to management scoring

For adherence, the mean scores per individual ranged from 10 to 40 and they were analysed as a continuous variable. Thus, the possible maximum and minimum adherence scores were 10 and 40, respectively.

### Long-term glycaemic control measures

The HbA1c machine provided a number from 4% to 13.8%. For clinical descriptive categorisation purposes, HbA1c≤7% was considered good control, while >7.1% was considered poor control (the higher the number, the poorer the glycaemic control).^43^ However, for regression and path analysis, we analysed long-term glycaemic as a continuous variable.

### Inferential statistical analysis

#### Univariate and multivariate regression analysis

We used regression analysis to find the strength and direction of the link between how patients felt about PCC, on the one hand and outcome variables such as self-efficacy, adherence and glycaemic control, on the other. The outcome variables were related to the patients’ sociodemographic and disease profile. Logistic regression was used for PCC since it was analysed as a binary variable (clusters), and, for self-efficacy, adherence and glycaemic control (HbA1c), we analysed as continuous variables using linear regression. We chose adherence as the main outcome variable because it is a cornerstone to the attainment of patient outcomes, mainly glycaemic control and the prevention or delay of DM complications^4 8 30^

In the light of the complex nature of the PCC construct and its covariates, and in order to further describe the effect of potential confounders in the pathway between PCC and sociodemographic characteristics, disease profiles, self-efficacy, adherence and DM complications, we used a multivariate logistic regression model adjusting for other variables; and the t-test with χ^2^ and p values for levels of significance at <0.05.^44^ A conceptual framework highlighting the possibility and direction of relationships between variables and their mediating factors guided the inferential statistical analysis. We reported unadjusted (OR) and adjusted (aOR) for logistic regression and unadjusted and adjusted beta (β) coefficients for linear regression. We ran an adjusted regression analysis for individual factors or subscales and the aggregate scale.

#### Path analysis to show the interrelationships between PCC, self-efficacy and adherence

To further clarify the magnitude and significance of predicted and observed relationships among the variables, we conducted a path analysis. A path model was iteratively built based on the theoretical hypothesis of the nature and directions of associations obtained from the initial regression output. The model was iteratively adjusted based on the magnitude of the modification indices and theoretical judgement.^44^ Our endogenous variables were PCC, efficacy, adherence and glycaemic control (as continuous variables), while exogenous variables included the sociodemographic and disease profile characteristics that were shown to affect the exogenous variable in the normal regression models. All endogenous variables were fed into the model as continuous variables. These exogenous variables included sex, age, tertiary education, type of diabetes and duration of disease. The path analysis β coefficients were used to judge the magnitude and direction of the predicted effect between the exogenous and the endogenous variables.

#### Model goodness-of-fit and ES

We determine the goodness of fit (GOF) for the binary regression models with a Hosmer-Lemeshow GOF χ^2^ value of >0.05 indicating that the regression models fitted well with the data.^45^ To determine if the hypothesised structural equation model fitted our data and the theoretical model, we assessed the following GOF indices of fit and their respective acceptable values; Comperative Fit Index (CFI): >0.9, Tucker Lewis Index (TLI)>0.9, Standardised Room Mean Squared Residual (SRMR)<0.08, Root Mean squared Error of Approximation (RMSEA)<0.05.

The R^2^ was used to determine how much of the effect on the endogenous variables is attributable to the predictor variables in the path analysis model. The R squared of >10% was deemed acceptable in empirical social science research as long as the linear relationships were statistically significant.^46^

## Results

### Participants’ sociodemographic characteristics and disease profile

The study population had significantly more females (n=390; 65%) than males. The mean age for males (51.6 years; SD 14.5) was significantly lower than that of females (54.5 years, SD 13.8). Nearly half (46.7 %) of the respondents had attained at least primary education. There were significantly more females (n=91, 23.3%) with no formal education compared with males (n=11, 50.3%). The majority of respondents had type 2 DM (n=520, 86.8%).

The respondents had their DM disease for an average duration of 6.9 years (SD 5.8) and had used the index facility for nearly the same period of time; 6.3 years (SD 5.5). The majority of patients (n=520, 86.7%) were type 2 diabetes and the most common comorbidities were hypertension (n=364, 60.8%), followed by HIV (n=93, 15.5%). Overall, there were significant levels of reported complications by the respondents. Numbness of the feet was the most common reported complication (n=465, 77.6%), followed by visual problems including blindness (n=315, 52.6%), and DM-related limb ulcers or amputations (n=161, 26.9). The study reported high levels of poor long-term glycaemic control, with 69.5% of the respondents having HbA1c >7%.

### The perception of PCC, self-efficacy and adherence scores

The unsupervised K-means cluster analysis highlighted two distinct groups of patients with different scores of PCC. Refer to online supplemental figure 1.

The first group had statistically significantly lower PCC scores than the first with mean scores of 51.6 (7.8) vs 77.0 (7.2) out of a possible score of 92 (p<0.001). More patients (n=234 out of 436) 55.7% of the patients belonged to the cluster with an average score of 51.56. Overall, slightly over half of the patients perceived low PCC during medical encounters, highlighting inconsistencies in the delivery of PCC. Women were more likely to experience low levels of PCC than men (aOR 0.48; 95% CI 0.86 to 0.10, p<0.05). Further segregation of the perception of PCC by age, disease type, duration of disease and facility did not show any significant differences (see table 1).

**Table 1:** The PCC clusters and their relationship to patient and disease profile and outcomes

Variable		PCC clusters			
	Characteristic	High	Low	Overall	P value*
Patient characteristics					
No. of patients	n (%)	193 (44.3.3	243 (55.8)	434 (100)	–
PCC scores	Mean (SD)	77.1 (7.2)	51.6 (7.8)	62.9 (14.8)	0.000
IQR (median)	IQR (median)	63-88 (79)	22-64 (80)	22-88 (61)	–
Sex (N %)	M	70 (36.3)	67 (27.6)	137 (31.4)	0.048
	F	123 (67.7)	126 (72.4)	299 (69.6)	–
Age (years)	Mean (SD)	53.5	53.7	53.6	0.912
Level of education n (%)	None	32 (16.6)	51(21)	83 (19.1)	0.243
	Primary	88 (45.6)	117 (49)	205 (47.2)	–
	Secondary	54 (28.0)	60 (25)	114 (26.0)	–
	Tertiary	19 (9.4)	15 (6.2)	33 (7.6)	–
Type of diabetes mellitus (%)	Type 1	26 (13.5)	33 (13.6)	59 (13.5)	0.004
	Type 2	167 (86.5)	210 (66.4)	377 (86.5)	–
Facility location N (%)	Urban	119 (61.7)	142 (58.4)	261 (75.4)	0.465
	Rural	74 (38.3)	101 (41.6)	175 (24.6)	–
Disease characteristics					–
Type of medicine n (%)	OHA	151 (78.0)	183 (75.3)	334 (77)	0.370
	OHA and Insulin	35(18)	43 (17.7)	78 (18)	–
and Insulin	7 (3.6)	15 (6.7)	22 (5.0)	–
	None	0 (0)	2 (0.8)	2 (0.04)	–
Duration of disease (years)	Mean (SD)	5.7 (4.9)	6.3 (5.5)	6.3 (5.2)	0.039
Duration of use of current facility (years)	Mean (SD)	6.4 (5.6)	7.3 (6.0)	7.0 (5.8)	0.112
Comorbidities: high BP	No	183 (94.8)	227 (93.2)	410 (94.0)	0.387
	Yes	10 (5.2)	16 (6.8)	26 (6.0)	–
Comorbidities: osteoarthritis	No	191 (98.1)	230 (94.5)	421 (96.6)	0.014
	Yes	2 (1.9)	13 (5.5)	15 (4.4)	–
Limb ulcers and amputations	No	163 (84.5)	140 (57.6)	303 (69.4)	0.000
	Yes	30 (15.5)	103 (42.4)	133 (29.6)	–
Vision problems and blindness	No	112 (58.0)	91 (37.4)	203 (46.6)	0.000
	Yes	81 (42.0)	152 (62.8)	233 (53.4)	–
Outcomes					–
Self-efficacy	Mean (SD)	54.9 (16.0)	57.6 (14.8)	56.1 (14.8)	0.015
Adherence	Mean (SD)	38.2 (1.6)	33.7 (2.4)	37.9 (2.1)	0.008
Glycaemic control	Poor	134 (69.4)	172 (70.8)	305 (88.2)	0.091
	Good	59 (30.6)	71 (29.2)	130 (11.8)	–

* t-test for continuous variables and χ2 test for categorical variables.

BP, blood pressure; OHA, Oral Hypoglycaemic Agents; PCC, patient-centred care; PREMs, Patient Reported Experience Measures ; PROMs, Patient Reported Outcome Measures .

The factors that were least perceived were patients’ involvement in care and organisation of care with a comparatively low score. The individualisation and involvement subscale had only 40% of patients experiencing the high mean scores compared with 55.7% for the global scale (online supplemental figure 2).

The efficacy scores ranged from 17 to 80 with a median score of 57 (mean 56.1 SD 14,6). The reported levels of adherence scores ranged from 23 to 40 with median 38 and mean of 37.8 (SD 2.1), respectively.

### Univariate and multivariate regression analysis

#### The perception of PCC

In the multivariate model, patients’ perception of PCC was significantly associated with adherence to management plans and serious DM complications with p values <0.05. Although the odds of perceiving low PCC were 1.49 higher if one were female compared with male (aOR 1.49; 95% CI 1.00 to 2.24, p<0.05) in the univariate model, after adjusting for other variables the relationship was no longer significant (refer table 2). The study also reported significantly higher odds of perceiving low PCC among patients who reported to have osteoarthritis (aOR 7.72, 95% CI 1.62 to 37.38, p<0.005) and also reported limb ulcers or amputations and comorbidities such as joint problems (aOR 4.67, 95% CI 0.2.66 to 4.67, p<0.001) compared with those who did not report these complications. There was no significant relationship between the perception of PCC with age, education status, the type of DM, type of medication and facility location (whether rural or urban).

**Table 2:** Univariate and multivariate regression analysis of the perception of PCC

Patient characteristics		Unadjusted OR			Adjusted OR		
		OR	P value	95% CI	aOR	P value	95% CI
Sex	Male (Ref)						
	Female	1.49	0.053	1.00 to 2.24	1.52	0.101	0.92 to 2.51
Age	Years	1.00	0.912	0.99 to 1.01	1.00	0.616	0.98 to 1.01
Level of education	None (Ref)						
	Primary	0.83	0.496	0.55 to 1.41	0.71	0.259	0.39 to 1.29
	Secondary	0.70	0.219	0.40 to 1.24	0.54	0.085	0.27 to 1.09
	Tertiary	0.50	0.089	0.22 to 1.11	0.40	0.070	0.15 to 1.08
Facility location	Urban (Ref)						
	Rural	1.14	0.496	0.78 to 1.68	0.98	0.948	0.62 to 1.57
Disease characteristics							
Type of diabetes mellitus	Type 1 (Ref)						
	Type 2	0.99	0.974	0.57 to 1.72	0.95	0.922	0.38 to 2.43
Type of medicine	OHA (Ref)						
	Insulin	1.01	0.957	0.62 to 1.66	1.37	0.469	0.58 to 3.22
	Insulin and OHA	1.77	0.226	0.70 to 4.45	2.15	0.144	0.77 to 6.03
Duration of facility use	Years	1.04	0.042	1.00 to 1.08	1.02	0.288	0.98 to 1.07
Comorbidities							
High blood pressure	No (Ref)						
	Yes	1.29	0.540	0.57 to 2.91	1.01	0.984	0.39 to 2.61
Osteoarthritis	No (Ref)						
	Yes	5.34	0.028	1.20 to 24.22	7.72	0.011	1.60 to 37.38
HIV on antiretroviral treatment	No (Ref)						
	Yes	0.67	0.483	0.22 to 2.04	0.48	0.355	0.10 to 2.29
Complications							
Numbness of feet	No (Ref)						
	Yes	1.54	0.070	0.97 to 2.46	1.14	0.656	0.65 to 2.01
Limb ulcers and amputations	No (Ref)						
	Yes	4.00	0.000	2.51 to 6.36	4.67	0.000	2.66 to 8.21
Vision problems and blindness	No (Ref)						
	Yes	2.31	0.000	1.60 to 3.40	1.52	0.078	0.95 to 2.43
Outcomes							
Self-efficacy	Score	1.02	0.016	1.00 to 1.03	1.02	0.016	1.00 to 1.04
Adherence	Score	0.88	0.009	0.79 to 0.97	0.75	0.000	0.66 to 0.85
Glycaemic control	Score	1.00	0.901	0.99 to 1.01	1.00	0.741	0.99 to 1.01

aOR, adjusted Odds ratio; OR, Odds Ratio; PCC, patient-centred care.

The odds of experiencing low PCC were 1.02 higher if your self-efficacy was high (aOR 1.02, 95% CI 1.00 to 1.04, p<0.005), and the odds lower if you were adherent (aOR 0.75, 95% CI 0.66 to 0.85, p<0.001).

Table 2 shows adjusted and unadjusted relationships between PCC and some sociodemographic characteristics, disease profile, comorbidities, self-efficacy, adherence and glycaemic control.

#### Self-efficacy

In the adjusted regression model, being female was associated with low self-efficacy with having a 2.99 unit decrease in self-efficacy scores (β=−2.99 95% CI −5.89 to −0.07, p<0.05) while having a tertiary level education was associated with an 8.76-point increase in efficacy scores (β=8.76, 95% CI 3.08 to 14.4, p<0.05). Reported visual complications are paradoxically associated with a 3.45-point increase in self-efficacy scores compared with none (β=3.45, 95% CI 0.66 to 6.25, p<0.05). The study did not find any relationship between age, facility location, type of DM and its treatment (see table 3).

**Table 3:** Showing univariate and multivariate analysis of adherence

		Unadjusted			Adjusted		
		β	P value	95% CI	β	P value	95% CI
Patient characteristics							
Sex	Male (Ref)						
	Female	−3.20	0.012	−5.70 to −0.70	−2.99	0.045	−5.89 to −0.07
Age (years)		−0.025	0.576	−0.11 to 0.06	–	–	–
Level of education	None (Ref)						
	Primary	1.54	0.376	−1.87 to 4.94	2.06	0.251	−1.47 to 5.60
	Secondary	4.78	0.011	1.12 to 8.45	3.61	0.075	−0.36 to 7.59
	Tertiary	7.86	0.004	2.56 to 13.16	8.76	0.003	3.08 to 14.44
Type of diabetes mellitus (%)	Type 1(Ref)						
	Type 2	3.79	0.039	0.19 to 7.39	1.67	0.553	−3.86 to 7.21
Facility location	Urban (Ref)						
	Rural	−0.78	0.539	−3.25 to 1.70	–	–	–
Disease characteristics							
Type of medicine	OHA (Ref)						
	Insulin	−3.86	0.018	−7.06 to −0.66	−1.12	0.660	−6.11 to 3.87
	Both Insulin and OHA	−6.19	0.028	−11.73 to −0.66	−4.97	0.115	−11.15 to 1.21
Duration of current facility (years)		0.23	0.051	−0.00 to 0.45	0.17	0.187	−0.08 to 0.41
Comorbidities: high BP	No (Ref)						
	Yes	0.07	0.953	−2.39 to 2.54			
Comorbidities: osteoarthritis	No (Ref)						
	Yes	−3.96	0.176	−9.70 to 1.78	–	–	–
Limb ulcers and amputations	No (Ref)						
	Yes	7.27	0.000	4.64 to 9.91	2.91	0.080	−0.35 to 6.16
Vision problems and blindness	No (Ref)						
	Yes	3.84	0.002	1.44 to 6.23	3.45	0.016	0.66 to 6.25
Outcomes							
Self-efficacy	Score						
Adherence	Score	2.03	0.000	1.48 to 2.58	2.00	0.000	1.32 to 2.69
Glycaemic control	Score	−1.21	0.000	−1.66 to −0.77	−0.96	0.000	−1.46 to −0.45
Perception of PCC	High (Ref)						
	Low	3.30	0.025	0.42 to 6.17	3.13	0.029	0.33 to 5.94

BP, blood pressure; PCC, patient-centred care.

The perception of low PCC was associated with a 3.13-point increase in self-efficacy scores (β=3.13 95% CI 0.33 to 5.94, p<0.05). Those that portrayed better adherence were also likely to have better self-efficacy (β=2.0, 95% CI 1.32 to 2.69, p<0.001) while better glycaemic control (lower HbA1c levels) was associated with a 0.96-point decrease in self-efficacy as well (β=−0.96, 95% CI −1.46 to −0.45, p<0.001). There was no significant relationship between self-efficacy and DM type, its treatment, comorbidities and other complications.

#### Adherence to DM management plans

In a univariate linear regression, having type 2 DM was associated with a 0.91 unit increase in adherence scores (β=0.91; 95% CI 0.13 to 1.70, p<0.05). Being female was significantly associated with an average increase of 0.31 in the odds of having better adherence scores than male counterparts (β=0.31; 95% CI 0.017 to 0.74, p<0.05). However, when we adjusted for other variables, the relationship was no longer significant. Likewise, those patients who were older and had better adherence scores with every increase in age (years) reported a corresponding average increase of 0.021 in adherence scores (β=0.03, 95% CI 0.013 to 0.03, p<0.001).

Having experienced low PCC levels in medical encounters was also significantly associated with poorer adherence, with a corresponding unit decrease of −0.88 in adherence score (β=−0.88; 95% CI −0.26 to −0.49, p<0.001) as PCC score goes down. Patients who portrayed better efficacy scores had better adherence, with every unit increase in self-efficacy score being relatto a 0.04 unit increase in adherence score (β=0.03, 95% CI 0.03 to 0.05, p<0.001). There was a marginal unit decrease of 0.07 in adherence scores if there were increased HbA1c values (β=−0.07; 95% CI −0.15 to −0.00, p<0.001). Thus, the better the adherence score, the better the glycaemic control. The study reported a decrease in adherence of 1.34 with taking a complicated regime (OHA—insulin combination compared with OHA alone) (β=−1.23, 95% CI −2.14 to −0.55, p<0.005). However, this relationship was not significant when controlled with other variables in a multivariate regression model. Notably, the level of education and hospital location had no significant relationship with being adherent to management care plans.

Table 4 shows adjusted and unadjusted relationships between adherence and some sociodemographic characteristics, disease profile, comorbidities, self-efficacy, PCC and glycaemic control.

**Table 4:** Showing univariate and multivariate analysis of adherence

		Unadjusted			Adjusted		
		β	P value	95% CI	β	P value	95% CI
Patient characteristics	Category						
Sex	Male (Ref)						
	Female	0.38	0.040	0.017 to 0.74	0.31	0.120	−0.08 to 0.70
Age (years)	Years	0.03	0.000	0.013 to 0.03	0.01	0.074	−0.00 to 0.03
Level of education	None (Ref)						
	Primary	−0.09	0.720	−0.57 to 0.41	–	–	–
	Secondary	−0.37	0.174	−0.90 to 0.16	–	–	–
	Tertiary	0.01	0.972	−0.76 to 0.79	–	–	–
Type of diabetes mellitus (%)	Type 1 (Ref)						
	Type 2	1.08	0.000	0.57 to 1.60	0.92	0.022	0.13
Disease characteristics							
Type of medicine	OHA (Ref)						
	Insulin	0-.75	0.001	−1.21 to −0.30	0.48	0.185	−0.23 to 1.19
	Both insulin and OHA	−1.34	0.001	−2.14 to −0.55	0.19	0.666	−0.66 to 1.03
Duration of disease (years)	Years	−0.01	0.403	−0.04 to 0.01	–	–	–
Comorbidities: high BP	No (Ref)						
	Yes	0.29	0.106	−0.06 to 0.65	–	–	–
Comorbidities: osteoarthritis	No (Ref)						
	Yes	−1.02	0.015	−1.85 to −0.20	−0.30	0.554	−1.30 to 0.70
Numbness of feet	No (Ref)						
	Yes	0.46	0.031	0.04 to 0.88	0.09	0.708	−0.38 to 0.56
Limb ulcers and amputations	No (Ref)						
	Yes	1.08	0.000	0.70 to 1.46	1.08	0.000	0.64 to 1.527181
Vision problems and blindness	No (Ref)						
	Yes	0.06	0.736	−0.29 to 0.41	–	–	–
Outcomes							
Self-efficacy	Score	0.04	0.000	0.03 to 0.05	0.04	0.000	0.03 to 0.05
Glycaemic control	Score	−0.16	0.000	−0.22 to −0.09	−0.07	0.049	−0.15 to −0.00
The perception of PCC	High (Ref)						
	Low	−0.41	0.045	−0.81 to −0.01	−0.88	0.000	−0.26 to −0.49

BP, blood pressure; PCC, patient-centred care.

#### Long-term glycaemic control

Notably, patients who had better glycaemic control were older, had better adherence and higher self-efficacy scores (p<0.01) in the unadjusted model although the relationship was no longer significant after controlling for other variables (see online supplemental table 5). The adjusted regression model reported better glycaemic control if you were older (β=−0.04, 95% CI −0.07 to −0.02, p<0.001). Thus, younger patients exhibited poorer glycaemic control than older patients. There was no significant change in glycaemic control among those who perceived high PCC compared with lower ones. Self-efficacy was the only independent predictor of glycaemic control with a marginal 0.04 unit decrease in HbA1c when efficacy scores increased (β=−0.04, 95% CI −0.06 to −0.02, p<0.001).

Although there was a 0.98 unit decrease in HbA1c if one had type 2 DM compared with type 1 DM (β=−0.81; 95% CI −1.63 to 1–0.33, p<0.005), after adjusting for other variables, the relationship was no longer significant. Likewise, the type of treatment and presence of complications seemed to correlate with glycaemic control in the unadjusted models, but the relationship was not significant after adjusting for other variables.

#### The analysis of individual subscales with self-efficacy, adherence and long-term glycaemic control

With regard to individual subscales, collaborative care was positively associated with adherence (β=0.66; 95% CI 0.04 to 0.09, p<0.05). There was no statistically significant relationship between the relational subscale and the organisational scale with adherence. Notably, there was a negative association between organisational subscale and self-efficacy (see online supplemental table 6). Thus, the predicted score of self-efficacy was reduced by a factor of 0.47% if the patient perceived better organisational aspects in their care (β=−0.47; 95% CI 0.79 to 0.18 p<0.001). There was no significant relationship between self-efficacy and perceiving better relational and collaborative aspects in their care. None of the subscales independently showed any significant relationship with glycaemic control. Refer to online supplemental figure 3 showing the analysis of individual subscales with self-efficacy, adherence and glycaemic control.

#### Path analysis: the relationship between PCC and other variables

The endogenous variables in this model included the perception of PCC, self-efficacy adherence and blood sugar level (HBA1c) while the exogenous variables included age, sex sugar type, presence of complications, presence of complications, concomitant hypertension and hospital location (urban vs rural). We had other endogenous variables predicting other endogenous variables such as adherence and self-efficacy in the model (see online supplemental figure 3).

The model was guided by observed relationships obtained from the initial regression model outputs and theoretical basis. It was iteratively adapted to get maximum GOF indices and theoretical plausibility. The statistical coefficients are shown in online supplemental table 7.

Online supplemental table 7 shows that female sex, presence of complications and comorbidities are significant negative predictors of the perception of PCC. There was a predicted 3.26 decrease in the perceiving PCC if one were female compared with male (β=−3.26, 95% CI −0.51 to –1.01, p<0.005). Having reported complications predicted an 8.4 decrease in the odds of perceiving better PCC (β=−4.62; 95% CI −6.87 to –2.38, p<0.001).

Although PCC was, theoretically, expected to predict better self-efficacy, we found no significant linear relationship between them (p>0.05). The presence of complications and tertiary education seemed to predict better efficacy in this study population. Having a tertiary education predicted a 6.4 increase in efficacy scores (β=6.36; 95% CI 1.96 to 10.76, p<0.05). Those on complicated management regimes were less self-efficacious with a predicted reduction of self-efficacy scores of 3.1 if one moves from OHA to more complicated regimens such as insulin and a combination of both (β=−3.71; 95% CI −5.1 to –1.23, p<0.05). Hospital location (urban vs rural) where patients accessed care did not seem to predict any significant differences in self-efficacy.

Both PCC and self-efficacy were positive predictors of adherence independently. Having perceived higher PCC predicted a marginal 0.03-point increase in adherence (β=0.03; 95% CI 0.01 to 0.04, p<0.005). The predicted effect of efficacy on adherence was of similar magnitude. Similarly, those that were older seemed to portray better adherence, with every unit increase in age predicting a 0.2 increase in adherence scores (β=0.02; 95% CI 0.03 to 0.03, p<0.05). Disaggregating PCC perception by factor revealed that the subscale which had patient engagement elements was associated with adherence to a greater degree (β=0.07; 95% CI 0.4 to 0.9, p<0.001) than the other two.

Self-efficacy and adherence were independent positive predictors of blood sugar control. Better self-efficacy predicted a 0.03 unit increase in the level of HbA1c (β=0.03; 95% CI 0.04 to 0.02, p<0.001). Additionally, higher adherence scores were associated with a 0.15 unit decrease in HbA1c (β=0.15; 95% CI 0.25 to 0.47, pe<0.05). Thus, better self-efficacy and adherence both independently predicted better glycaemic control. The younger one is, the worse the glycaemic control, with a predicted increase of 0.03 in HbA1c as one gets younger. Thus, younger patients who also had type one diabetes had predicted poor glycaemic control generally.

### Model GOF and variance

The model we used for path analysis statistically fit our data well in predicting endogenous variables using multiple GOF indices. The GOF indices showed the following values: RMESA: 0.37, CFI: 930 and TLI: 880 and SRMR: 0.25. The model was able to explain 30% of the variance in the observed data (see online supplemental table 8).

Regarding the dependent observable variables, namely PCC, self-efficacy, adherence and blood sugar control, the overall R^2^ was 0.30, indicating the variance explained by the model. Therefore, the model was able to explain 30% of the relationships in this prototype. The endogenous variables in the model were PCC, self-efficacy, adherence and blood levels which were able to explain 14%, 17% and 10% of the relationships, respectively, in our study population. The R^2^ was low at just about 8% for self-efficacy. There could be other endogenous variables not considered in this model that may explain the portrayed relationships, especially for self-efficacy and glycaemic control.

## Discussion

Using the contextual operational framework, we found that most patients perceived low PCC as defined by fearful interactional ambience, less individualisation and involvement, and a poor organisation of care context. The extent of the poor PCC perception standing at 55.7% in this study was comparable with the Ethiopian studies (Bahir City and Addis Ababa) which found the perception of poor PCC at 53.7% and 51%, respectively.^47^ ^48^ Both these studies showed significantly poorer PCC in public health facilities compared with private ones. This is possibly due to comparable resource challenges in the two contexts, where HCPs hardly have time to interact with their patients except to finish the queue.^17^ Malawi’s proportions of poor PCC (55.7%) are considerably higher compared with the SA study (16%).^47^ This is possibly due to the differences in the type of scales and the method used to dichotomise the scores. In the SA study, the dichotomisation of PCC scores was largely qualitative, while in our study, the categorisation of the scores was statistical and scientific with an unsupervised K-means clustering technique. Furthermore, it indicated cut-off points that made a statistical difference in eliciting management outcomes.^49^ This adds value to the construct validation of the tool, which this study considers a strength because it further dictates the level at which PCC would matter for improving outcomes.

Principally, the result highlights the inadequacies and inconsistencies in the delivery of PCC by providers during medical encounters, most deficient in the individualisation and patient involvement subscales. This finding is further corroborated by De Man *et al*, who in their systematic review of literature from sub-Saharan Africa lament the overly biomedical orientation of medical training and incentivisation in most African healthcare delivery systems.^1^ Medical training and healthcare delivery systems hardly value PCC approaches; therefore, most medical interactions in African contexts are overly paternalistic, with no due attention given to quality partnership building.^28^ While most studies mention organisational, structural and patient barriers as accounting for low PCC experiences in medical encounters, the biomedical orientation of service delivery, lack of time and overworked providers stand out in low resource contexts.^17 28^

The presence of complications and comorbidities predicts a negative perception of PCC. In their study, Kuipers *et al* show that people with multimorbidities and complications have compromised physical comfort and are therefore likely to report having perceived less PCC, especially if it does not respond to their current physical needs.^50^ Conversely, the results highlight the need to tailor PCC to those that need it most and the extent to which they need it because it matters in the realisation of PREMS and PROMS. The presence of complications and comorbidities comes with both emotional and physical discomfort, creating more complex patients’ care needs, hence the perceived need for more tailored PCC.^51^ The finding confirms that PCC, by its nature, should help HCPs unearth patients’ health needs and be responsive to them in unique ways that are satisfying to each patient’s situation. Concurrently, in our study population, there is a predominance of women who also have complications, which can explain the preponderance for women to perceive less PCC, just as Kuipers *et al* found in their study.^50^ The fact that location (rural vs urban), education status, sugar type and duration of disease did not affect the perception of PCC confirms that PCC is a need for all patients’ regardless of a social background; only the emphasised elements may evolve across visits.

The individualisation and involvement subscale were notably the least experienced, with lower mean scores than factors (relational and organisational) comparatively. Although patients perceived that their provider’s interactions were a little bit more relational, the human connection during the encounter was not adequate enough to create an environment for invitation and support for participation in care. Patients’ experiences of being acknowledged as unique individuals and involved in care in our study population are gloomy.^52^ Malawi alludes to the same in its quality management policy (QMD) where deficiencies in patient involvement in care were identified.^27^ Fearful interactional ambience, power imbalances, insufficient dialogue and patients’ own restrictive attitudes towards engagement with their providers deter patient involvement.^52^ There is a need for an adequate build-up from the creation of a good relational ambience to acknowledging patients, tailoring their care to their unique experiences and empowering and supporting them towards self-care behaviours.^53^ The human connection process, therefore, ought to manage power imbalances and patient vulnerabilities related to patient involvement. It makes sense, therefore, that without the enabling interactional environment in the encounters, patient involvement would hardly happen.

The perceptions of PCC as being positively associated with adherence are not surprising. PCC interactions in medical encounters have the potential to build health literacy and support and capacitate patients towards adherence to self-care behaviours.^8 54^ Thus, adherence is, in part, a marker of effective provider–patient interaction that has facilitated trust building, information sharing, patient involvement and support. Additionally, adherence is a cornerstone to the attainment of patient outcomes, glycaemic control and the prevention or delay of DM complications.^55^ The finding validates that PCC is not just a quality issue; it also impacts intermediate outcomes, including glycaemic control. Especially when patients are active partners in their care, they are better able to adhere to mutually agreed on goals of care. While we understand that glycaemic control and the reduction of complications is multifactorial, adherence to care management plans is an effective starting point if we are to achieve some gains in DM patient outcomes.

Surprisingly, the relationship between PCC and self-efficacy was not statistically significant in the path analysis. As a social cognitive resource, self-efficacy, especially as it relates to specific health-related self-care activities, requires a significant level of support during medical encounters through verbal persuasion by the HCP or peers, acknowledgement and validation of patients’ capabilities, and mastery of the activities.^56 57^ The general absence of empowering and supportive elements in the perceived PCC in this study may explain the non-significant relationship to start with. PCC goes beyond good relational ambience or friendliness to include support towards mastery of self-care competencies with the provider as a resource.^58^ Therefore, in contexts where there’s marked power imbalances between the patients and their providers, poor medical literacy, and patient self-confidence is low or is hardly harnessed, PCC supportive efforts during medical encounters need to acknowledge, validate and leverage patients’ capabilities in self-care for the attainment of better outcomes.^22 57 59^ The absence of a relationship between the PCC and self-efficacy may, in part, also be attributable to the fact that the study did not include other related or mediating constructs such as social support, health literacy and outcome expectations (person’s estimate that a given behaviour will lead to certain outcomes) which also impact self-efficacy.^22 34 60^

Self-efficacy and adherence independently predict better long-term glycaemic control in our study, which resonates with most studies.^4 60 61^ Haskard Zolnierek and Dimatteo, in their meta-analysis, highlight how PCC provider behaviours can enhance adherence by helping patients understand their illness, the treatment and its benefits, enhance collaborative partnerships and support for self-care.^4^ Thus, PCC that prioritises patient involvement works by capacitating patients to adhere to their treatment plans, thereby yielding better glycaemic control.^7 11 30^ Even though in our study PCC does not predict better self-efficacy, we see that self-efficacy itself is a predictor of better glycaemic control. According to social cognitive theory, self-efficacy has the potential to impact outcomes independently.^22 60 61^ In addition, our study found better glycaemic control among older patients compared with younger ones. Likely, with increasing age comes experience and mastery of self-care activities. Tertiary educational status being a predictor of self-efficacy in our study population is not surprising, as it may be regarded as a proxy for health literacy, an important element in the development of self-efficacy behaviours.^62^

Over two-thirds (70%) of the patients had poor long-term glycaemic control reported. It may be that there is general poor glycaemic control reported in this study and other studies emanate from other reasons, such as the chronic shortage of drugs alluded to through the qualitative phase of this study context.^63^^(p. 108)^ Within the study population, we concurrently found that access to DM medication was a yearned-for and yet lacking aspect of PCC. Thus, even though PCC predicts some marginal increases in adherence, patients may only be taking their drugs as and when they are available. Likewise, there may be more determinants of glycaemic control that this study did not elicit apart from adherence to drugs and lifestyle.^54^ Therefore, we advocate that PCC delivery must spill over beyond the interactional realm to stimulate larger organisational improvements if we are to make a significant difference in patient outcomes. Notably, therefore, in implementing PCC, healthcare delivery systems ought to strive to address the basic organisational issues such as access to medication and timeliness of care which matter to patients with DM.

The study confirms that the perception of PCC has potential to predict outcomes such as adherence and glycaemic control. The pathway linking PCC to glycaemic control is not direct but, rather, through adherence. The absence of a link between PCC and self-efficacy in this study has been attributed to the general absence of empowerment aspects of PCC in the study, which capacitates patients to feel confident in themselves to undertake self-care activities. The finding still validates the need for chronic PCC encounters to be supportive and empowering towards mastery of self-care.

The study recorded strengths and limitations that need to be borne in mind while making conclusions and applying the findings. The use of a locally developed and validated tool to measure the perception of PCC is novel and ensured contextual relevance of the tool. Again, the use of multiple and complementary analytical statistical methods in assigning patients into distinct subgroups (parallel analysis and unsupervised K-means clustering) ensured triangulation of findings, and the dichotomisation of PCC scores into high and low was scientific and used cut-offs that mattered in the determination of patient outcomes. The finding seems to suggest a probable minimum level of PCC that is required to mediate patient outcomes. Further interventional studies are required to validate this proposition.

Given the complex nature of PCC as a social behavioural construct, the use of both logistical regression into a path analysis ensures that the effect and direction of associations of significant covariates are well considered in the analysis models. The hierarchical nature in the employment of statistical methods ensured that the results were scientific and reliable.

Despite the above, the study records some shortcomings. First, being a cross-sectional, non-interventional study, it falls short of describing causal relationships between PCC and related outcomes. Second, PCC being a sociobehavioural phenomenon, has other determinants that may explain the relationships between itself and outcomes that were not considered in this study, such as social support and health literacy. Hence, some aspects that may mediate the associations between PCC and outcomes may not have been measured, thereby limiting the study’s ability to explain some associations or absence of others with certainty.

Notwithstanding these limitations, the results have highlighted potential relationships within the PCC-DM management pathway that can be further studied. This is significant because this is the first time a Malawian study has objectively assessed how patients perceive PCC; elucidating some patterns in the PCC-DM management-outcome pathway. Therefore, the findings have set a platform for subsequent exploration.

### Implications for practice

This baseline evaluation of patient-centredness and timeliness measures contributes to our understanding of the current state of quality of care. Our operational framework can inform the development of PCC interventions and care innovation models that effectively improve medication adherence and patient outcomes in DM care. Medical encounters need to foster interactions that improve patient involvement, leveraging on patient capabilities thereby improving adherence to management care plans. We recommend further interventional studies using this validated framework to elucidate the feasibility of such studies and the causal relationship of PCC and outcomes.

## Conclusions

While PCC is not an end in itself, the initial patterns show that it is a potential intervention in the improvement of DM care, especially for patients with or at risk of complications. Above and beyond creating a conducive ambience, medical encounters ought to evoke support and capacitation for patients to undertake self-care behaviours confidently. While it is obvious that patients with DM in Malawi perceive poor PCC, we must ride on the political commitment that is currently within the country’s Ministry of Health’s documented policies to advance these interpersonal aspects of quality of care because they matter. PCC largely remains providers’ responsibility, hence the urgent need to invest in medical education that cultivates PCC competencies and attitudes. Appraising and incentivising PCC elements will help improve its advocacy.

## Supplementary material

10.1136/bmjopen-2024-090308 online supplemental file 1

## Data Availability

Data are available on reasonable request. All data relevant to the study are included in the article or uploaded as supplementary information.

## References

[1] De Man J, Mayega RW, Sarkar N (2016). Patient-Centered Care and People-Centered Health Systems in Sub-Saharan Africa: Why So Little of Something So Badly Needed?. IJPCM.

[2] Hudon C, Fortin M, Haggerty JL (2011). Measuring patients’ perceptions of patient-centered care: a systematic review of tools for family medicine. Ann Fam Med.

[3] McMillan SS, Kendall E, Sav A (2013). Patient-Centered Approaches to Health Care. Med Care Res Rev.

[4] Zolnierek KBH, Dimatteo MR (2009). Physician communication and patient adherence to treatment: a meta-analysis. Med Care.

[5] Little P, Everitt H, Williamson I (2001). Observational study of effect of patient centredness and positive approach on outcomes of general practice consultations. BMJ.

[6] Mead N, Bower P, Hann M (2002). The impact of general practitioners’ patient-centredness on patients’ post-consultation satisfaction and enablement. Soc Sci Med.

[7] Rathert C, Wyrwich MD, Boren SA (2013). Patient-centered care and outcomes: a systematic review of the literature. Med Care Res Rev.

[8] Robinson JH, Callister LC, Berry JA (2008). Patient-centered care and adherence: definitions and applications to improve outcomes. J Am Acad Nurse Pract.

[9] World Health Organization (2003). Adherence to longterm therapies: evidence to action.

[10] Miles A (2010). Tailoring care to individuals and populations within resource-poor settings: A review and commentary on the World Health Organisation Report People-Centred Care in Low and Middle Income Countries. IJPCM.

[11] Michie S, Miles J, Weinman J (2003). Patient-centredness in chronic illness: what is it and does it matter?. Patient Educ Couns.

[12] Bem Daryl J (1972). Self perception theory. Adv Exp Soc Pyschol.

[13] Rathert C, Williams ES, McCaughey D (2015). Patient perceptions of patient‐centred care: empirical test of a theoretical model. Health Expect.

[14] Stewart M (2001). Towards a global definition of patient centred care. The patient should be the judge of patient centred care. BMJ.

[15] Clayton MF, Latimer S, Dunn TW (2011). Assessing patient-centered communication in a family practice setting: how do we measure it, and whose opinion matters?. Patient Educ Couns.

[16] Budhwani S (2013). The Self-Management-Focused Chronic Care Model: A Conceptual Framework. In: CAHSPR Conference. Toronto: Health system performance network. https://ihpme.utoronto.ca/.

[17] Makwero M, Muula A, Anyawu FC (2021). The conceptualisation of patient-centred care: A case study of diabetes management in public facilities in southern Malawi. Afr j prim health care fam med.

[18] Castro EM, Van Regenmortel T, Vanhaecht K (2016). Patient empowerment, patient participation and patient-centeredness in hospital care: A concept analysis based on a literature review. Patient Educ Couns.

[19] Louw JM, Marcus TS, Hugo JFM (2017). Patient- or person-centred practice in medicine? - A review of concepts. Afr J Prim Health Care Fam Med.

[20] World Health Organisation - Western Pacific Region (2007). People centred health care: a policy framework.

[21] Nayiga S, DiLiberto D, Taaka L (2014). Strengthening patient-centred communication in rural Ugandan health centres: A theory-driven evaluation within a cluster randomized trial. Evaluation (Lond).

[22] Reisi M, Mostafavi F, Javadzade H (2016). Impact of Health Literacy, Self-efficacy, and Outcome Expectations on Adherence to Self-care Behaviors in Iranians with Type 2 Diabetes. Oman Med J.

[23] Black N, Varaganum M, Hutchings A (2014). Relationship between patient reported experience (PREMs) and patient reported outcomes (PROMs) in elective surgery. BMJ Qual Saf.

[24] World Health Organization, World Bank Group, OECD (2018). Delivering quality health services: a global imperative for universal health coverage.

[25] Government of Malawi (2018). The malawi non communicable disease & injuries poverty commision report; reframing non communicable diseases for the poorest billion.

[26] Waweru E, Sarkar NDP, Ssengooba F (2019). Stakeholder perceptions on patient-centered care at primary health care level in rural eastern Uganda: A qualitative inquiry. PLoS ONE.

[27] Government of the Republic of Malawi (2017). Quality management policy for the health sector in Malawi.

[28] Cubaka VK, Schriver M, Kayitare JB (2018). He should feel your pain. Africa J Prim Healthc Fam Med.

[29] Althubaiti A (2023). Sample size determination: A practical guide for health researchers. *J Gen Fam Med*.

[30] Ratner NL, Davis EB, Lhotka LL (2017). Patient-Centered Care, Diabetes Empowerment, and Type 2 Diabetes Medication Adherence Among American Indian Patients. Clin Diabetes.

[31] Wejnert C, Pham H, Krishna N (2012). Estimating design effect and calculating sample size for respondent-driven sampling studies of injection drug users in the United States. AIDS Behav.

[32] Şadiye Y, Ünsal A (2017). Hemşirelikte Araştırma Geliştirme Dergisi The Validity and Reliability Study of Self-Efficacy Scale on the People with Chronic Dieases * Kronik Hastalıklarda Öz - Etkililik Ölçeğinin Geçerlik ve Güvenirlik Çalışması.

[33] Kerari A (2023). The psychometric properties of the Diabetes Self-Efficacy Scale in Saudis with type 2 diabetes. *Nurs Open*.

[34] Kwanjo Banda C, Gombachika BT, Nyirenda MJ (2019). Self-management and its associated factors among people living with diabetes in Blantyre, Malawi: a cross-sectional study. *AAS Open Res*.

[35] Stanford Patient Education Research Center (2009). Self-Efficacy for Diabetes. National Institute of Nursing Research (NINR).

[36] Lambert EV, Steyn K, Stender S (2006). Cross-cultural validation of the hill-bone compliance to high blood pressure therapy scale in a South African, primary healthcare setting. Ethn Dis.

[37] Song Y, Han H, Jung SH (2011). Psychometric Evaluation of Hill-Bone Medication Adherence Subscale. Asian Nurs Res (Korean Soc Nurs Sci).

[38] Commodore-Mensah Y, Delva S, Ogungbe O (2023). A Systematic Review of the Hill-Bone Compliance to Blood Pressure Therapy Scale.

[39] Msyamboza KP, Mvula CJ, Kathyola D (2014). Prevalence and correlates of diabetes mellitus in Malawi: population-based national NCD STEPS survey. BMC Endocr Disord.

[40] Cohen DB, Allain TJ, Glover S (2010). A survey of the management, control, and complications of diabetes mellitus in patients attending a diabetes clinic in Blantyre, Malawi, an area of high HIV prevalence. Am J Trop Med Hyg.

[41] Mohammed MB, Zulkafli HS, Adam MB (2021). Comparison of five imputation methods in handling missing data in a continuous frequency table. https://pubs.aip.org/aip/acp/article-abstract/2355/1/040006/684231/Comparison-of-five-imputation-methods-in-handling?redirectedFrom=fulltext.

[42] Sawyer SF (2009). Analysis of Variance: The Fundamental Concepts. J Manual Manipulative Ther.

[43] Aschner P (2017). New IDF clinical practice recommendations for managing type 2 diabetes in primary care. Diabetes Res Clin Pract.

[44] Lancaster T, Duncan OD (1976). Introduction to Structural Equation Models. J R Stat Soc Ser A.

[45] Voinov V (2013). Chi-squared goodness of fit tests with applications.

[46] Ozili PK (2022). The Acceptable R-Square in Empirical Modelling for Social Science Research The Acceptable R-square in Empirical Modelling for Social Science Research. SSRN Electron J.

[47] Ewunetu M, Temesgen W, Zewdu D (2023). Patients’ Perception of Patient-Centered Care and Associated Factors Among Patients Admitted in Private and Public Hospitals: A Comparative Cross-Sectional Study. Patient Prefer Adherence.

[48] Birhanu Z, Assefa T, Woldie M (2010). Determinants of satisfaction with health care provider interactions at health centres in central Ethiopia: a cross sectional study. BMC Health Serv Res.

[49] Morissette L, Chartier S (2013). The k-means clustering technique: General considerations and implementation in Mathematica The k -means clustering technique: General considerations and implementation in Mathematica. Tutor Quant Methods Psychol.

[50] Kuipers SJ, Cramm JM, Nieboer AP (2019). The importance of patient-centered care and co-creation of care for satisfaction with care and physical and social well-being of patients with multi-morbidity in the primary care setting. BMC Health Serv Res.

[51] Hopman P, Schellevis FG, Rijken M (2016). Health-related needs of people with multiple chronic diseases: differences and underlying factors. Qual Life Res.

[52] Makwero M, Muula AS, Anyanwu FC (2022). An insight into patients’ perspectives on barriers affecting participation in shared decision making among patients with diabetes mellitus in Malawi. *BMC Prim Care*.

[53] Thórarinsdóttir K, Kristjánsson K, Krist T (2014). Patients’ perspectives on person-centred participation in healthcare: a framework analysis. Nurs Ethics.

[54] Williams JS, Walker RJ, Smalls BL (2016). Patient-Centered Care, Glycemic Control, Diabetes Self-Care, and Quality of Life in Adults with Type 2 Diabetes. Diabetes Technol Ther.

[55] Hamm C (2006). Improving care for people with long-term conditions.

[56] Irie K, Bandura ARV (2021). The routledge handbook of the psychology of language learning and teaching.

[57] Warner LM, Schwarzer R (2020). Self‐Efficacy and Health. Wiley Encycl Heal Psychol.

[58] Warner LM, Ziegelmann JP, Schüz B (2011). Maintaining autonomy despite multimorbidity: self-efficacy and the two faces of social support. Eur J Ageing.

[59] Schwarzer R, Leppin A (1991). Social Support and Health: A Theoretical and Empirical Overview. J Soc Pers Relat.

[60] Martos-Méndez MJ (2015). Self-efficacy adn adherence to treatment: The mediating effects of social support. J of Beh Hea & Soc Iss.

[61] Okuboyejo S, Mbarika V, Omoregbe N (2018). The effect of self-efficacy and outcome expectation on medication adherence behavior. *J Public Health Afr*.

[62] Chen AMH, Yehle KS, Albert NM (2014). Relationships between health literacy and heart failure knowledge, self-efficacy, and self-care adherence. Res Social Adm Pharm.

[63] Government of the Republic of Malawi (2013). Malawi Service Provision Assessment Survey (MPSA) 2013-14: key findings.

