# Supplementary material for: Perception of patient-centred care and its relationship with management outcomes and complications among patients with DM in Malawi
Source: BMJ Open. 2025 Jul 5;15(7):e090308. doi: 10.1136/bmjopen-2024-090308 (PMC12228445; doi:10.1136/bmjopen-2024-090308)
Supplement: online supplemental file 1 [file bmjopen-15-7-s001.docx]

# The perception of patient-centred care and its relationship with management outcomes and complications among patients with DM in Malawi

*Martha Makwero^1, 2^, Adamson S. Muula^2^, Felix Chima Anyanwu^1^, Innocent Maposa^1^, Jude Igumbor^1^*

*^1^ School of Public Health, Faculty of Health Sciences, University of the Witwatersrand, South Africa*

*^2^* *Department of Family Medicine, University of Malawi, Kamuzu University of Health Sciences, Malawi*

*Corresponding Author: Dr Martha Makwero.*

*Department of Family Medicine, University of Malawi, Kamuzu University of Health Sciences, Malawi, Private Bag 3360, Blantyre, Malawi*

*Email: mmakwero@cartafrica.org,* [*mmakwero@kuhes.ac.mw*](mailto:mmakwero@kuhes.ac.mw)

*Phone: +265 884111312.*

*Keywords: Patient-centred care, perception, adherence, self-efficacy, glycaemic control*

Contents

[The perception of patient-centred care and its relationship with management outcomes and complications among patients with DM in Malawi 1](#_Toc15798)

[Figure 1. Cluster Plot 2](#_Toc30185)

[Figure 2:  Showing the statistics for the global PCC scale and individual subscales  2](#_Toc29165)

[Table 5: Univariate and multivariate regression analysis for glycaemic control 4](#_Toc29492)

[Table 6:  The analysis individual subscales with self-efficacy, adherence and glycaemic control 6](#_Toc9688)

[Figure 3: Path analysis model showing the statistical and theoretical relationship among variables  6](#_Toc26422)

[Table 7: Showing the nature of relationships, coefficients and p-values between variables 6](#_Toc24879)

[Table 8: The variance for Equation level goodness of fit  7](#_Toc6971)

## Figure 1. Cluster Plot


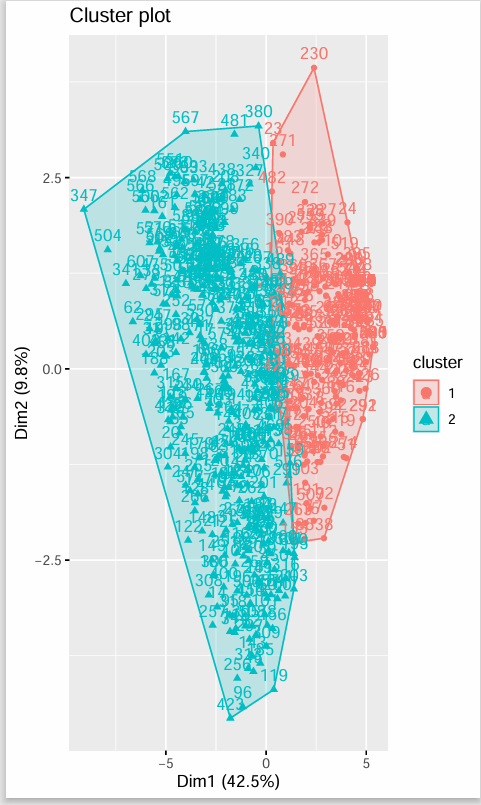


## Figure 2:  Showing the statistics for the global PCC scale and individual subscales


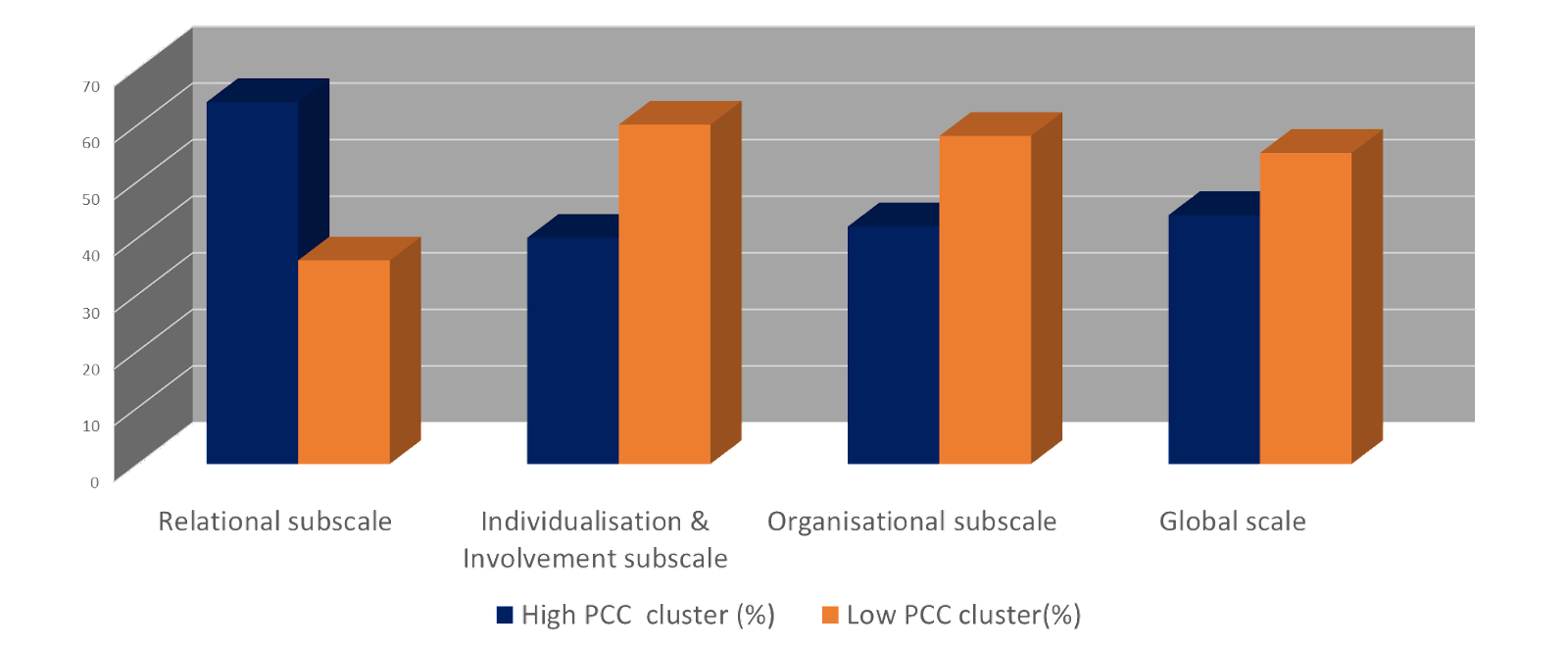


## Table 5: Univariate and multivariate regression analysis for glycaemic control

|  |  | **UNADJUSTED** | | | | **ADJUSTED** | | | |
| --- | --- | --- | --- | --- | --- | --- | --- | --- | --- |
|  |  | **β** | **p-value** | **95% CI** | | **β** | **p-value** | **95% CI** | |
| **PATIENT CHARACTERISTICS** | | |  |  |  |  |  |  |  |
| **SEX** | Male (Ref) | |  |  |  |  |  |  |  |
|  | Female | -0.120 | 0.605 | -0.57 | 0.33 | 0.17 | 0.525 | -.36 | 0.70 |
| **AGE (YEARS)** |  | -0.035 | 0.000 | -0.05 | -0.02 | -0.04 | 0.000 | -0.07 | -0.02 |
| **LEVEL OF EDUCATION** | None (Ref) |  |  |  |  |  |  |  |  |
|  | Primary | 0.42 | 0.185 | -0.20 | 1.04 | - | - | - | - |
|  | Secondary | 0.25 | 0.460 | -0.42 | 0.92 | - | - | - | - |
|  | Tertiary | 0.55 | 0.264 | -0.42 | 1.52 | - | - | - | - |
| **TYPE OF DIABETES MELLITUS (%)** | Type 1(Ref) | |  |  |  |  |  |  |  |
|  | Type 2 | -0.98 | 0.003 | -1.63 | -0.33 | 0.80 | 0.139 | -0.26 | 1.85 |
| **DISEASE CHARACTERISTICS** |  |  |  |  |  |  |  |  |  |
| **TYPE OF MEDICINE** | OHA (Ref) |  |  |  |  | - | - | - | - |
|  | Insulin | 1.16 | 0.000 | 0.59 | 1.74 | 0.48 | 0.319 | -0.47 | 1.42 |
|  | OHA & Insulin | 1.10 | 0.029 | 0.11 | 2.10 | 0.25 | 0.674 | -0.90 | 1.39 |
| **DURATION OF DISEASE** | Years | 0.04 | 0.046 | 0.00 | 0.08 | 0.05 | 0.016 | 0.01 | 0.10 |
| **DURATION OF USE CURRENT FACILITY** | Years | 0.04 | 0.060 | -0.00 | 0.08 | - | - | - | - |
| **COMORBIDITIES: HIGH BP** | No (Ref) |  |  |  |  |  |  |  |  |
|  | Yes | -0.81 | 0.000 | -3.63 | -1.25 | -0.38 | 0.188 | -0.96 | 0.19 |
| **COMORBIDITIES: OSTEOARTHRITIS** | No (Ref) |  |  |  |  |  |  |  |  |
|  | Yes | -0.47 | 0.376 | -1.51 | 0.57 | - | - | - | - |
| **NUMBNESS OF FEET** | No (Ref) |  |  |  |  |  |  |  |  |
|  | Yes | 0.05 | 0.845 | -.47 | 0.58 | - | - | - | - |
| **LIMB ULCERS & AMPUTATIONS** | No (Ref) |  |  |  |  |  |  |  |  |
|  | Yes | -0.15 | 0.549 | -0.64 | 0.34 | - | - | - | - |
| **VISION PROBLEMS & BLINDNESS** | No (Ref) |  |  |  |  |  |  |  |  |
|  | Yes | 0-.02 | 0.942 | -0.45 | 0.42 | - | - | - | - |
| **OUTCOMES** |  |  |  |  |  |  |  |  |  |
| **SELF-EFFICACY** | Score | -0.04 | 0.000 | -0.05 | -0.03 | -0.04 | 0.000 | -0.06 | -0.02 |
| **ADHERENCE** | Score | -0.24 | 0.000 | -0.35 | -0.15 | -0.12 | 0.084 | -0.25 | 0.02 |
| **PERCEPTION OF PCC** | High (Ref) |  |  |  |  |  |  |  |  |
|  | Low | -0.16 | 0.525 | -0.67 | 0.34 | -0.28 | 0.290 | -0.80 | 0.24 |

## Table 6:  The analysis individual subscales with self-efficacy, adherence and glycaemic control

|  | Self-efficacy | | | | Adherence | | | | Glycaemic control | | | |
| --- | --- | --- | --- | --- | --- | --- | --- | --- | --- | --- | --- | --- |
| subscale | β | P-value | 95% Conf Interval | | β | P-value | 95% Conf Interval | | β | P-value | 95% Conf Interval | |
| Relational ambience | 0.08 | 0.96 | -2.36 | 2.51 | 0.34 | 0.59 | -0.01 | 0.07 | -1.62 | 0.39 | -5.29 | 2.04 |
| Individualization and involvement | -1.69 | 0.16 | -4-06 | 0.69 | 0.043 | 0.16 | 0.078 | 0.77 | --0.77 | 0.67 | -4.35 | 2.81 |
| Organization | -5.85 | 0.00 | -8.17 | -3.35 | -0.03 | 0.86 | -0.38 | -0.32 | 1.29 | 0.467 | -2.27 | 4.85 |

## Figure 3: Path analysis model showing the statistical and theoretical relationship among variables


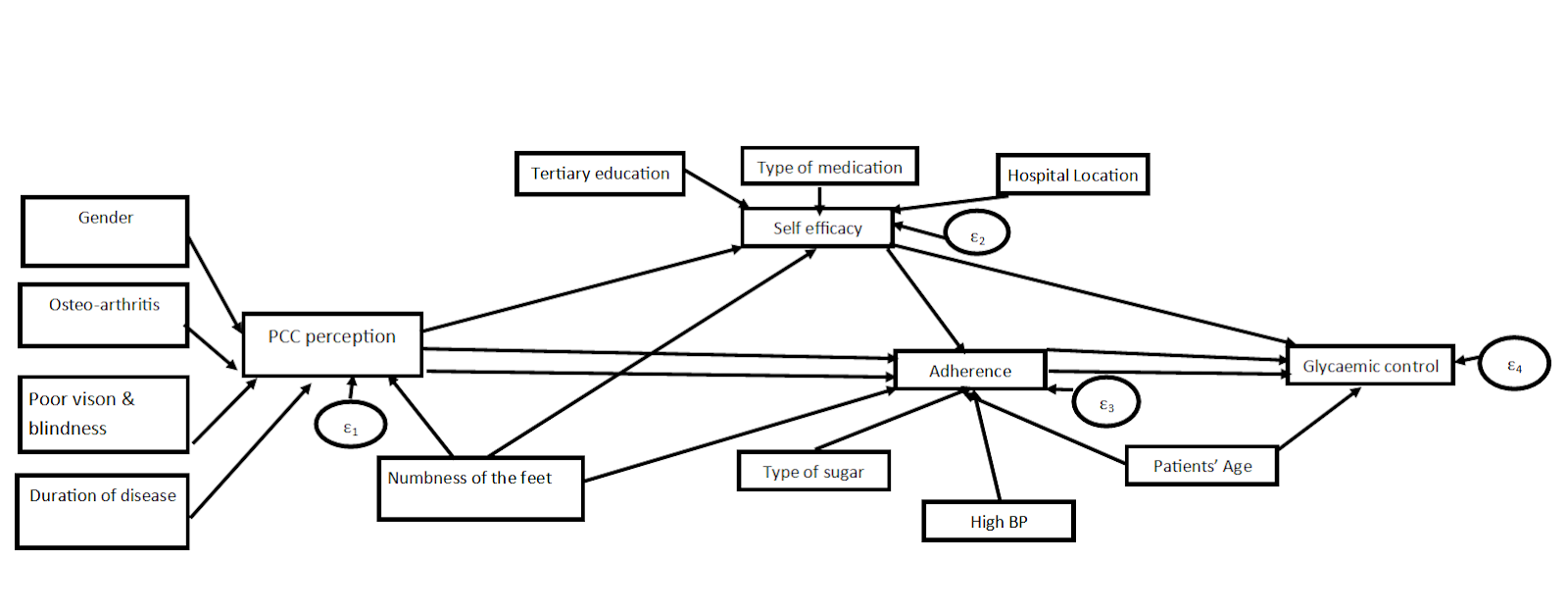


## Table 7: Showing the nature of relationships, coefficients and p-values between variables

| **ENDOGENOUS VARIABLES** | **EXOGENOUS VARIABLES** | **BETA COEFFICIENT** | **P-VALUE** | **95% CONFIDENCE INTERVAL** | |
| --- | --- | --- | --- | --- | --- |
| **PERCEPTION OF PATIENT CENTRED CARE (PCC)** | Gender(female) | -3.26 | 0.004 | -0.51 | -1.01 |
|  | Comorbidities (knee problems) | -7.38 | 0.005 | -12.50 | -2.27 |
|  | Complications (Limb ulcers and amputations) | -4.62 | 0.000 | -6.87 | -2.38 |
|  | Complications (kidney problems) | -8.18 | 0.000 | -10.72 | -5.65 |
|  | Duration of diseases | -0.17 | 0.112 | -0.37 | 0.04 |
| **SELF-EFFICACY** | PCC | 0.02 | 0.693 | -0.07 | 0.10 |
|  | Complications (Limb ulcers and amputations) | 7.31 | 0.000 | 4.56 | 10.06 |
|  | Level of education (Tertiary) | 6.36 | 0.005 | 1.96 | 10.76 |
|  | Medication type | -3.18 | 0.001 | -5.08 | -1.27 |
|  | Hospital location | -2.30 | 0.062 | -4.72 | 0.12 |
| **ADHERENCE** | PCC | 0.03 | 0.000 | 0.01 | 0.04 |
|  | self-efficacy | 0.04 | 0.000 | 0.02 | 0.05 |
|  | Complications (Limb ulcers and amputations) | 0.93 | 0.000 | 0.54 | 1.31 |
|  | Age | 0.02 | 0.010 | 0.00 | 0.03 |
|  | Type of sugar (Type 2) | 0.49 | 0.079 | -0.06 | 1.03 |
|  | Comorbidities (hypertension) | 0.49 | 0.168 | -0.21 | 1.19 |
| **LONG-TERM GLYCAEMIC CONTROL** | PCC | 0.01 | 0.347 | 0.02 | 0.01 |
|  | self-efficacy | 0.03 | 0.000 | 0.05 | 0.02 |
|  | Adherence | -0.15 | 0.007 | -0.25 | -0.04 |
|  | Age | 0.03 | 0.000 | 0.06 | 0.02 |

## Table 8: The variance for Equation level goodness of fit

|  |  |  |  |  |  |  |
| --- | --- | --- | --- | --- | --- | --- |
| **VARIABLE** | **Variance** | | |  | **Correlation** | |
|  | **Fitted** | **Predicted** | **Residual** | **R-squared (%)** | **Dep Vs. Pred** | **B-T corr.** |
| **THE PERCEPTION OF PATIENT CENTRED CARE (PCC)** | 201.58 | 29.61 | 171.97 | 14.68 | 0.38 | 0.15 |
| **SELF-EFFICACY** | 211.70 | 17.50 | 194.20 | 8.27 | 0.29 | 0.08 |
| **ADHERENCE** | 4.39 | 0.74 | 3.64 | 16.97 | 0.41 | 0.17 |
| **LONG-TERM GLYCAEMIC CONTROL** | 6.96 | 0.68 | 6.28 | 9.80 | 0.31 | 0.10 |
| **OVERALL** |  |  |  | **30.07** | - | - |

**Appendix 1: The Malawi Patient centred care Tool**


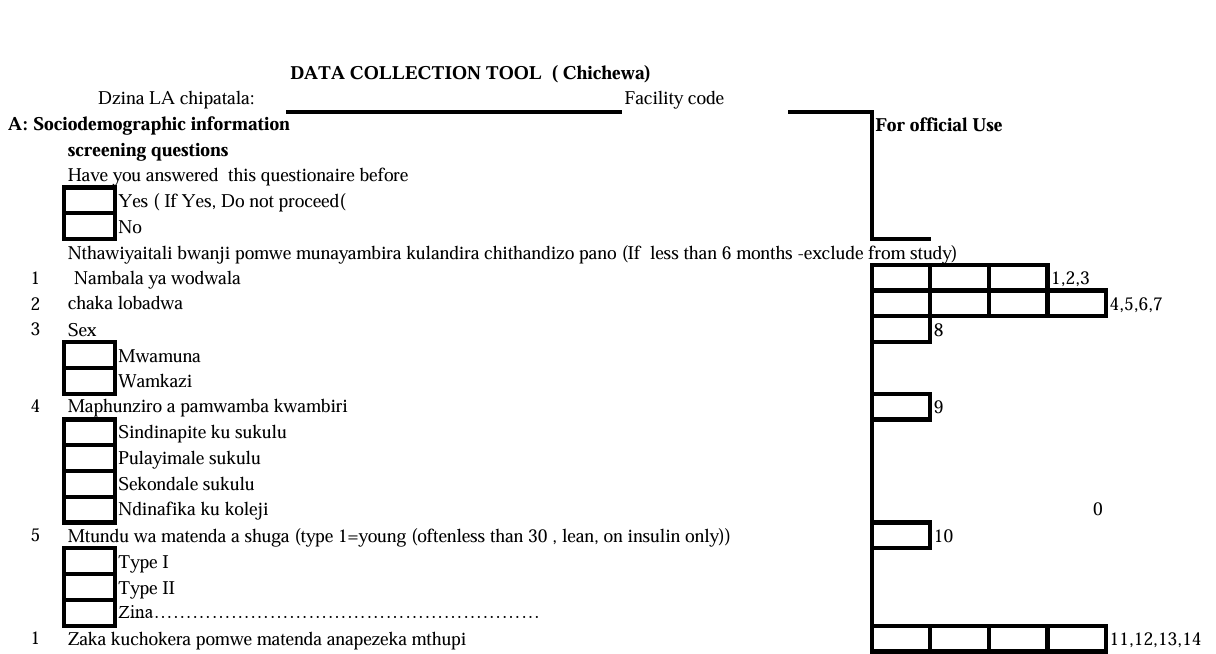


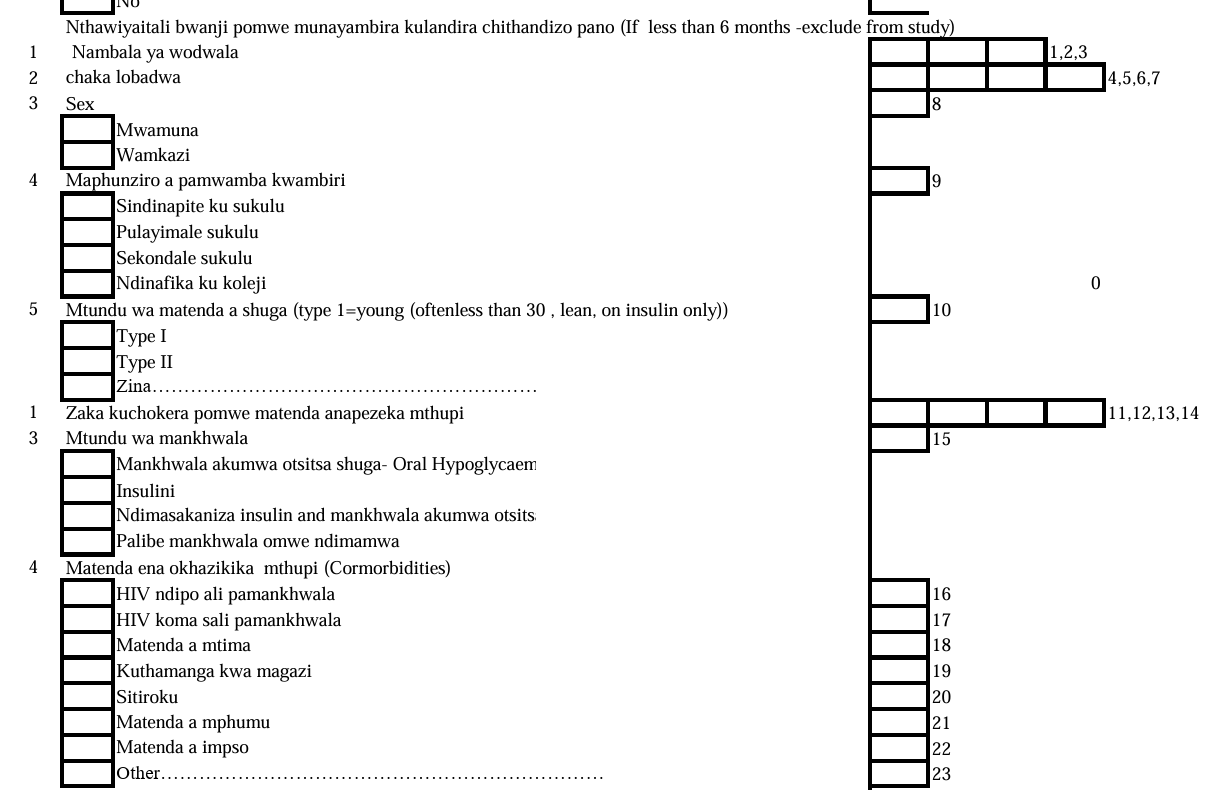


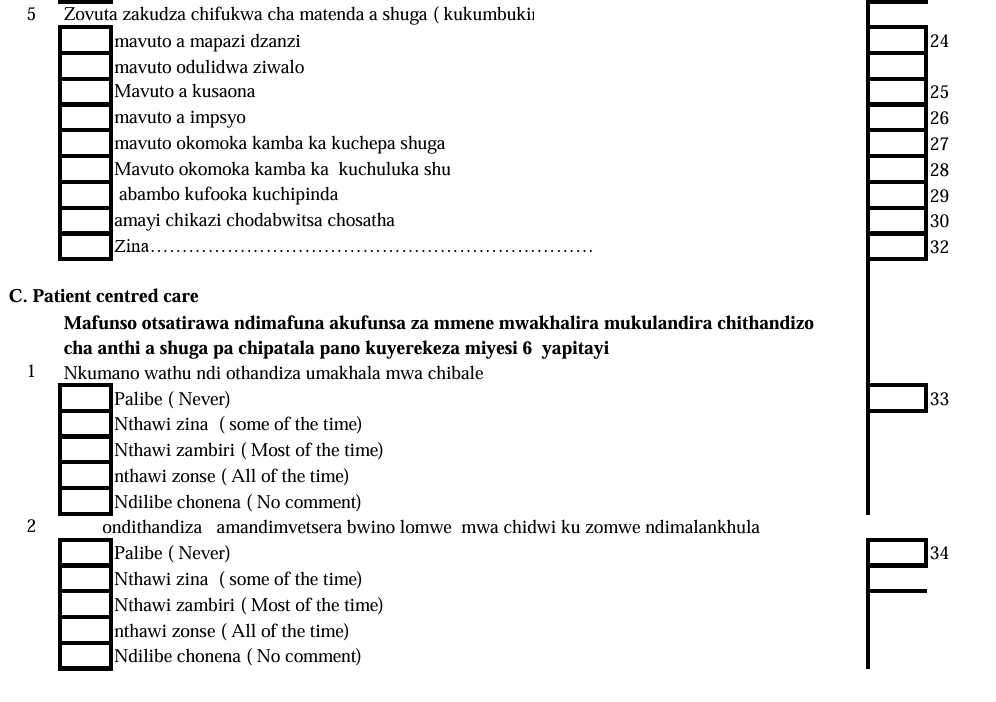


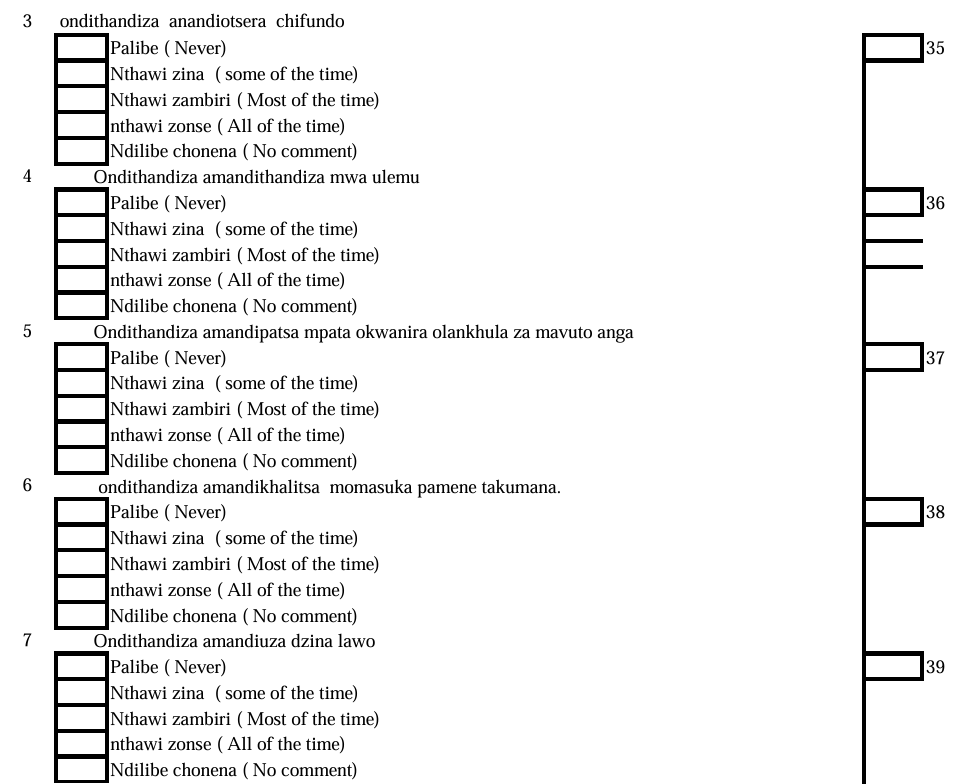


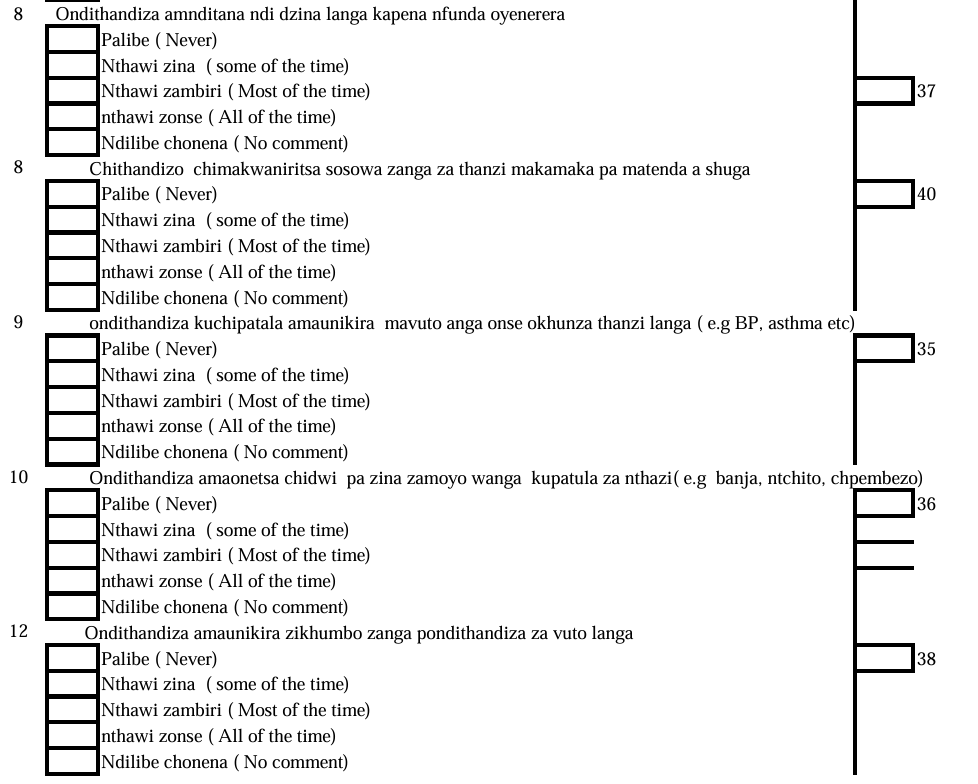


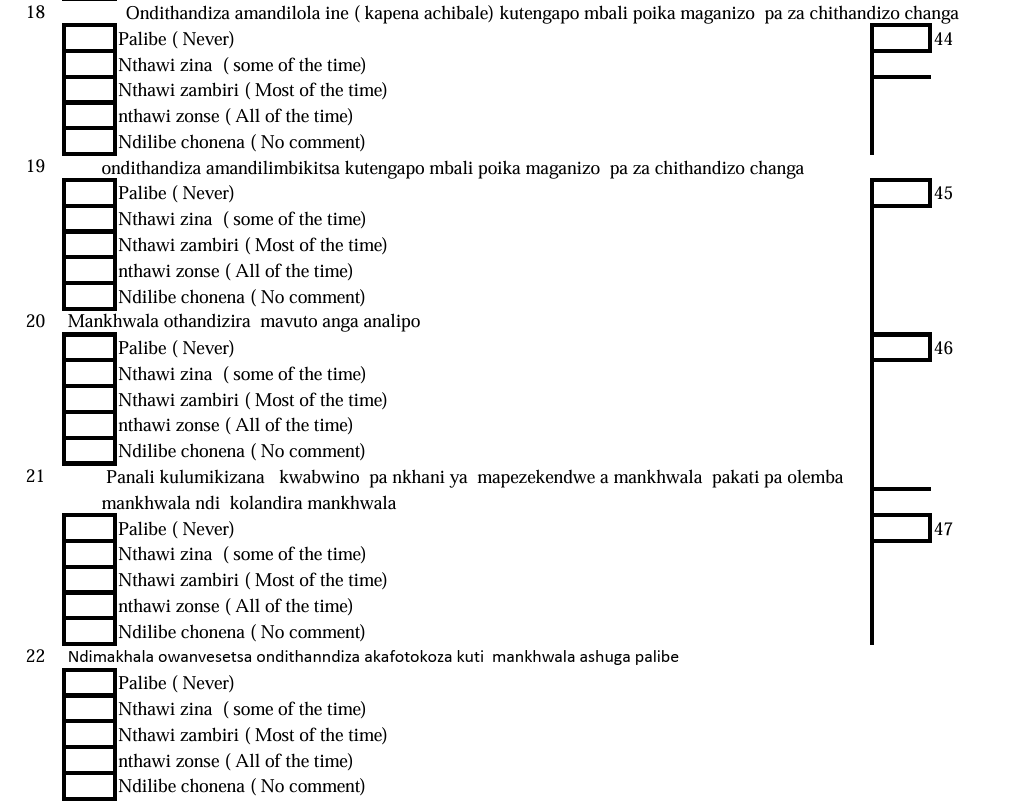


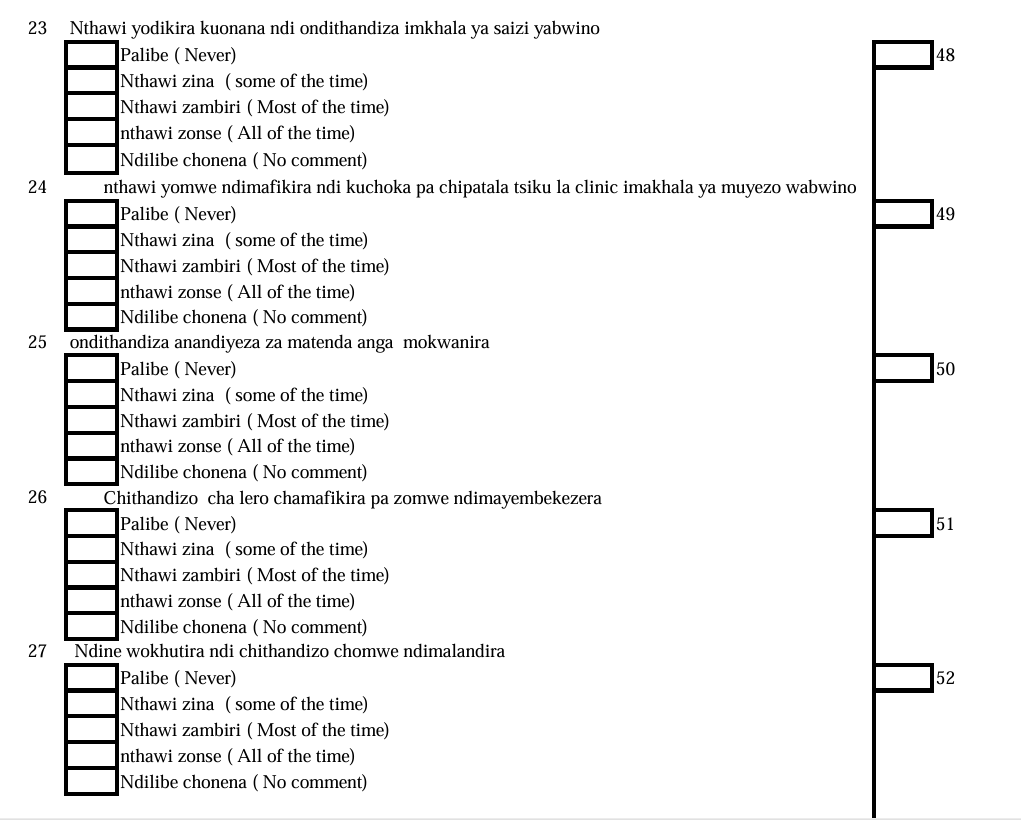


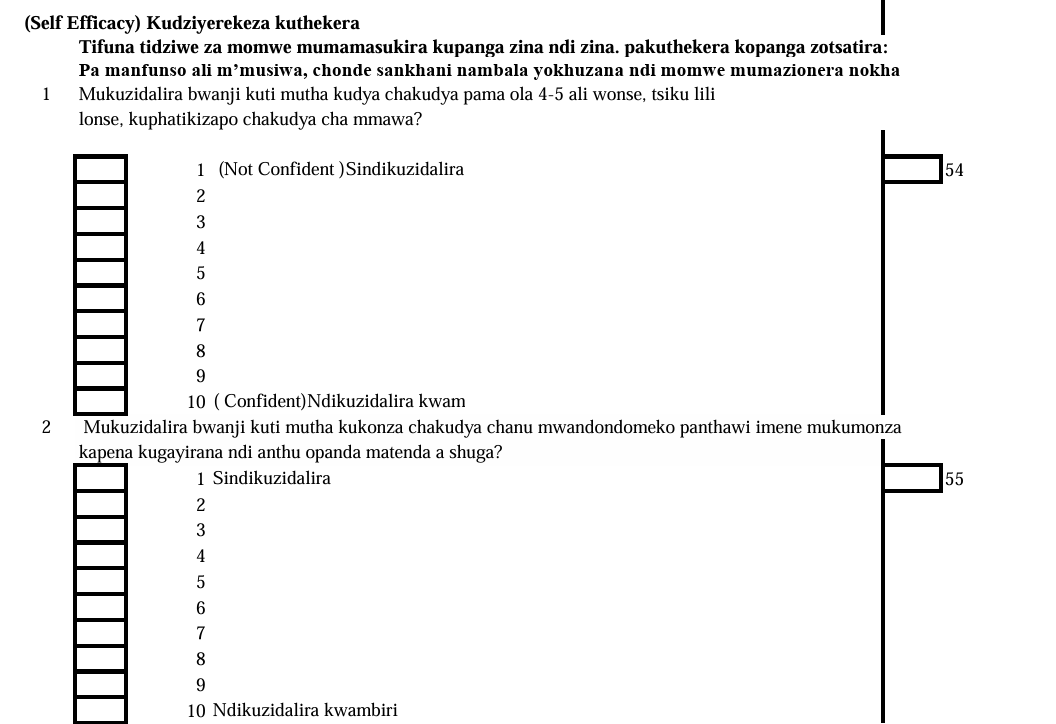


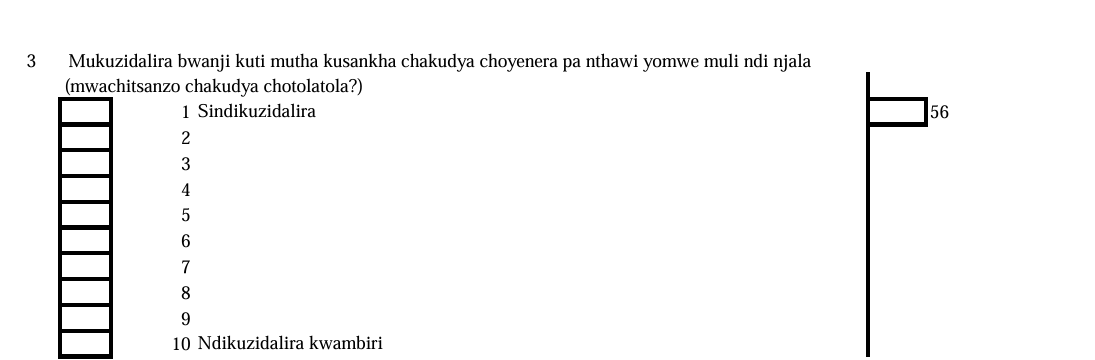


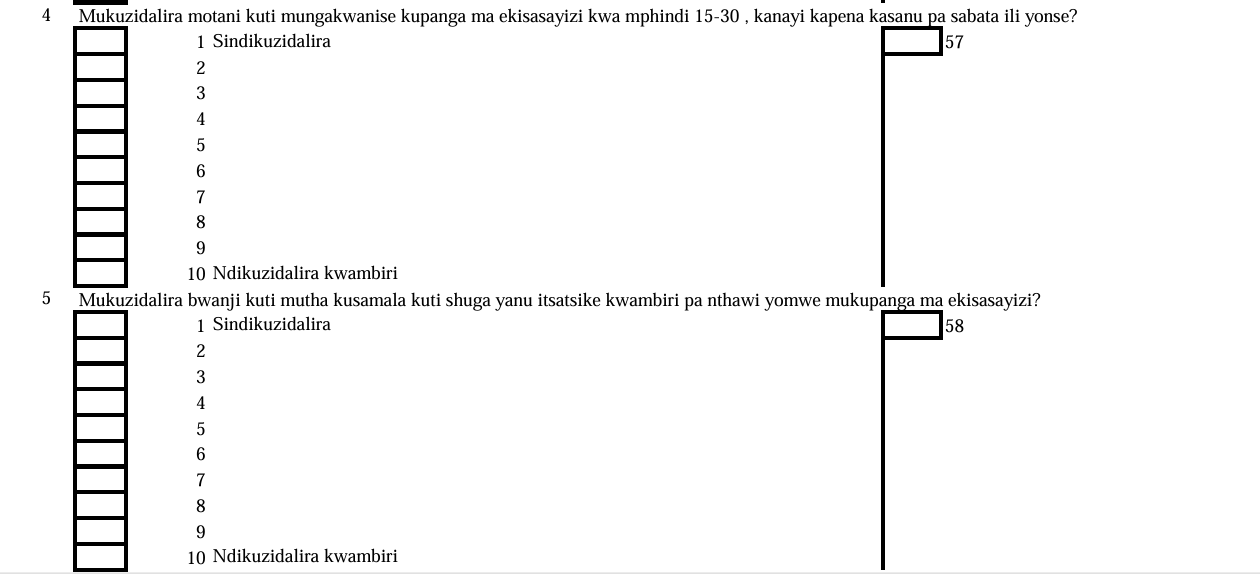


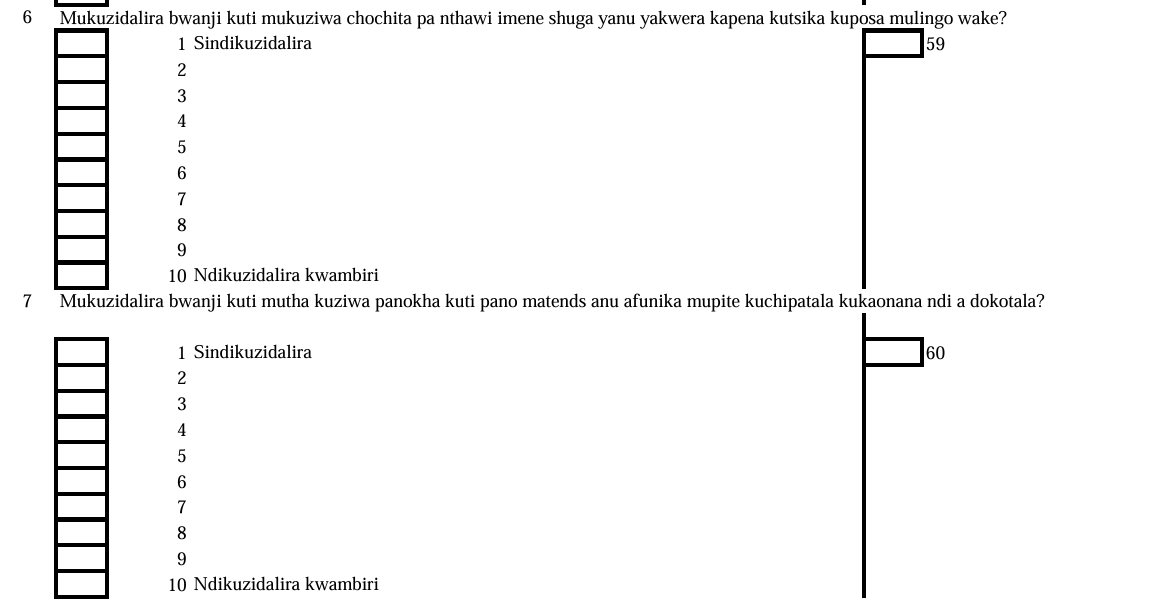

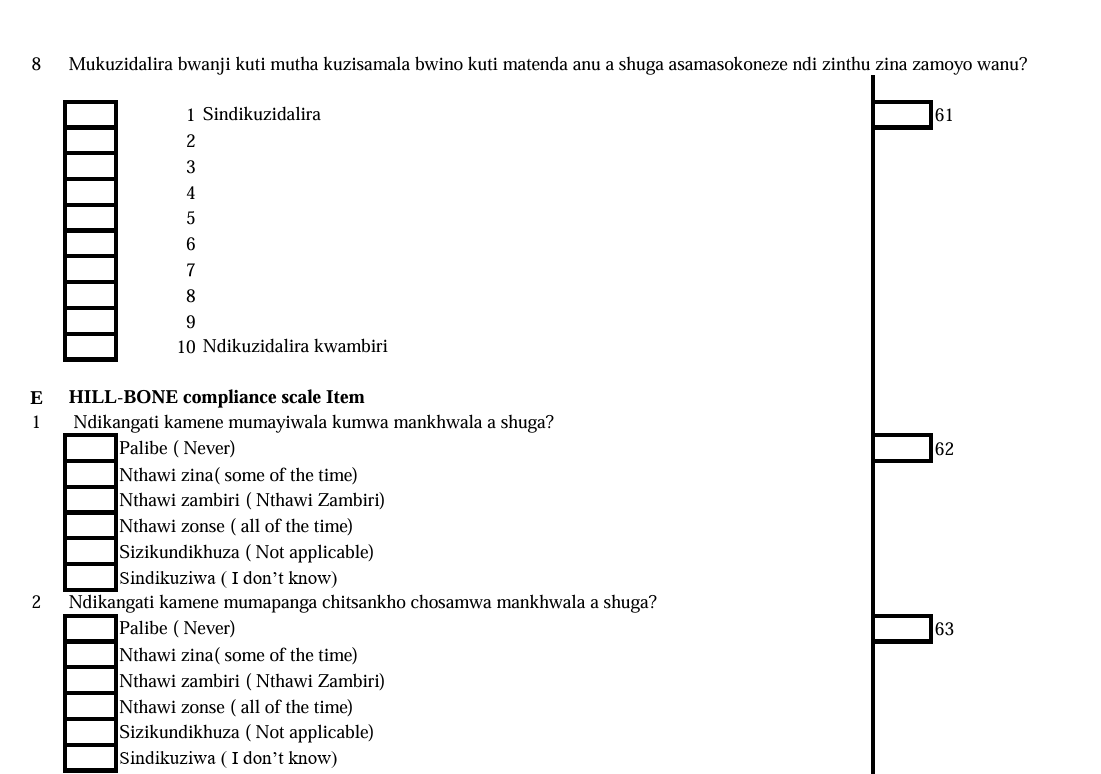


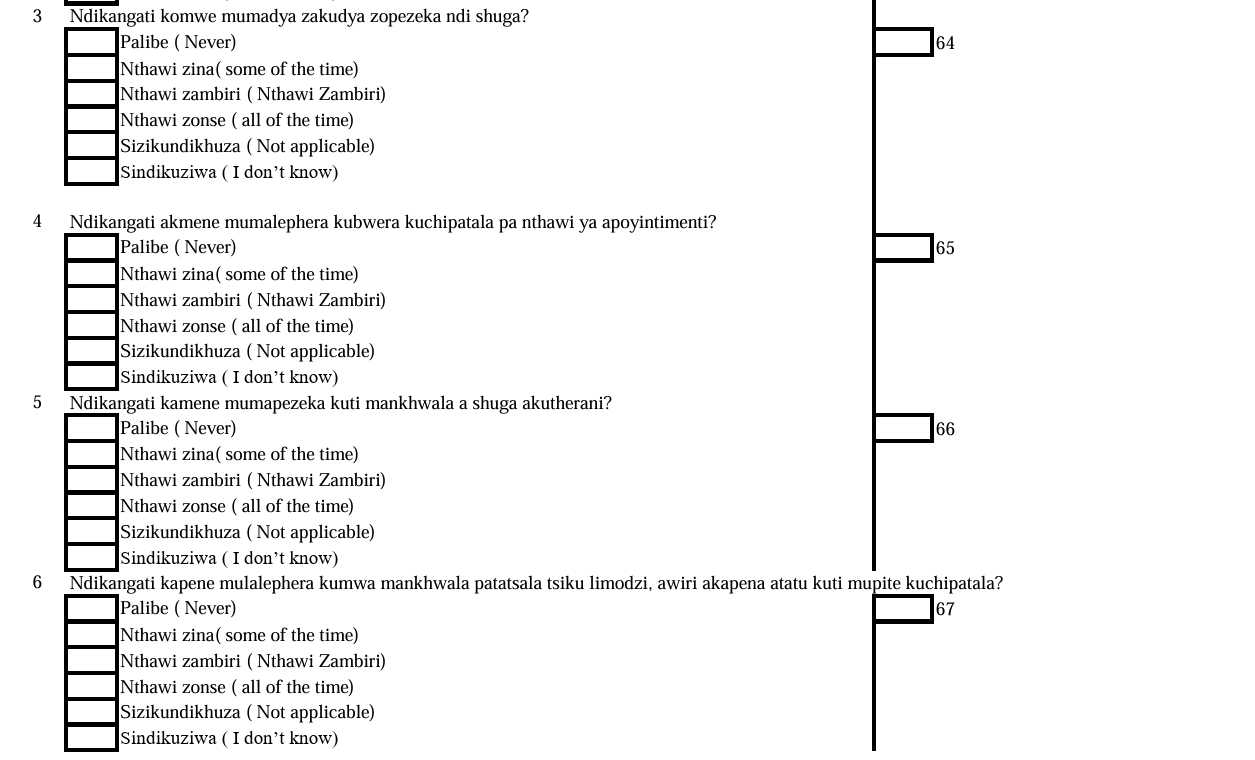


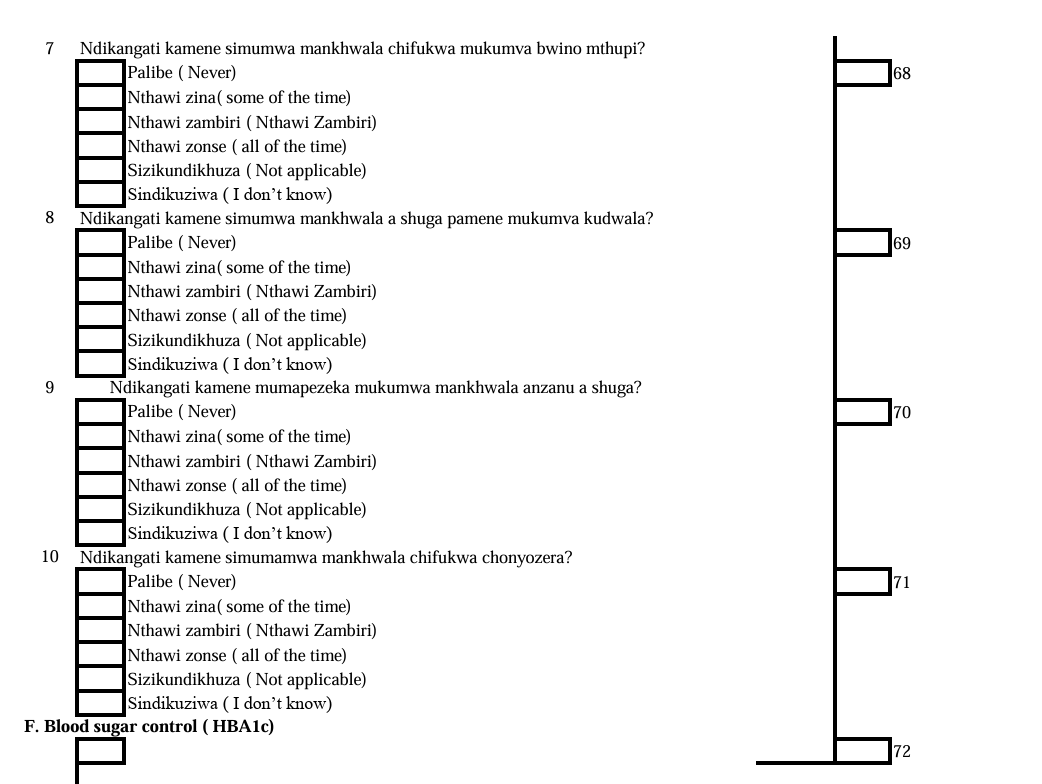


**Appendix 2: Stanford self-efficacy tool**

We would like to know how confident you are in doing certain activities. For each of the following questions, please choose the number that corresponds to your confidence that you can do the tasks regularly at the present time.

|  | \| How confident do you feel that you can eat your meals every 4 to 5 hours every day, including breakfast every day? \| \| --- \| | 1  Not confident | 2 | 3 | 4 | 5 | 6 | 7 | 8 | 9 | 10  Totally confident |
| --- | --- | --- | --- | --- | --- | --- | --- | --- | --- | --- | --- | --- |
|  | \| How confident do you feel that you can follow your diet when you have to prepare or share food with other people who do not have diabetes? \| \| --- \| | 1 | 2 | 3 | 4 | 5 | 6 | 7 | 8 | 9 | 10 |
|  | \| How confident do you feel that you can choose the appropriate foods to eat when you are hungry (for example, snacks)? \| \| --- \| | 1 | 2 | 3 | 4 | 5 | 6 | 7 | 8 | 9 | 10 |
|  | \| How confident do you feel that you can exercise 15 to 30 minutes, 4 to 5 times a week? \| \| --- \| | 1 | 2 | 3 | 4 | 5 | 6 | 7 | 8 | 9 | 10 |
|  | \| How confident do you feel that you can do something to prevent your blood sugar level from dropping when you exercise? \| \| --- \| | 1 | 2 | 3 | 4 | 5 | 6 | 7 | 8 | 9 | 10t |
|  | \| How confident do you feel that you know what to do when your blood sugar level goes higher or lower than it should be? \| \| --- \| | 1 | 2 | 3 | 4 | 5 | 6 | 7 | 8 | 9 | 10 |
|  | \| How confident do you feel that you can judge when the changes in your illness mean you should visit the doctor? \| \| --- \| | 1 | 2 | 3 | 4 | 5 | 6 | 7 | 8 | 9 | 10 |
|  | \| How confident do you feel that you can control your Diabetes so that it does not interfere with the things you want to do \| \| --- \| | 1 | 2 | 3 | 4 | 5 | 6 | 7 | 8 | 9 | 10 |

## Appendix 3: Hill Bone adherence scale

| **HILL-BONE compliance scale Item** | | None of the time | Some of the time | Most of the time | All of the time | Not applicable | Don’t Know |
| --- | --- | --- | --- | --- | --- | --- | --- |
| 1 | How often do you forget to take your DM medicine? |  |  |  |  |  |  |
| 2 | How often do you decide not to take your DM medicine? |  |  |  |  |  |  |
| 3 | How often do you take sugary food? |  |  |  |  |  |  |
| 4 | How often do you miss scheduled appointments? |  |  |  |  |  |  |
| 5 | How often do you run out of DM pills? |  |  |  |  |  |  |
| 6 | How often do you skip your DM medicine 1–3 days before you go to the clinic? |  |  |  |  |  |  |
| 7 | How often do you miss taking your DM pills when you feel better? |  |  |  |  |  |  |
| 8 | How often do you miss taking your DM pills when you feel sick? |  |  |  |  |  |  |
| 9 | How often do you take someone else’s DM pills? |  |  |  |  |  |  |
| 10 | How often do you miss taking your DM pills when you do not feel like taking them |  |  |  |  |  |  |

## Appendix 4: Ethical approval certificates

### Appendix 4.1: The Malawi College of Medicine Research Ethics committee ethical approval certificate


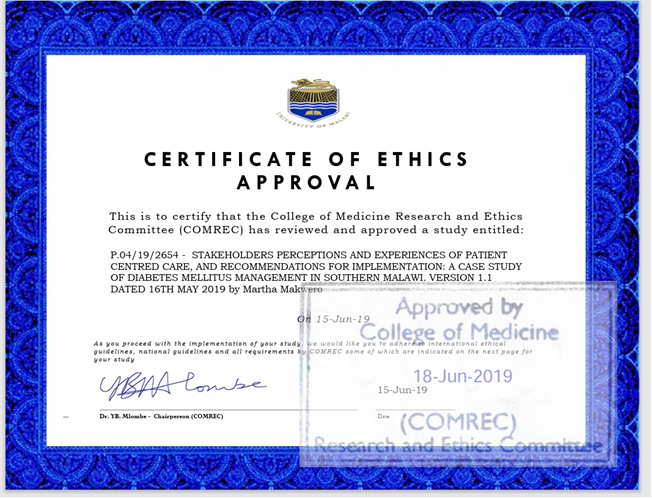


### Appendix 4.2 Witwatersrand Health Research (HREC) approval committee


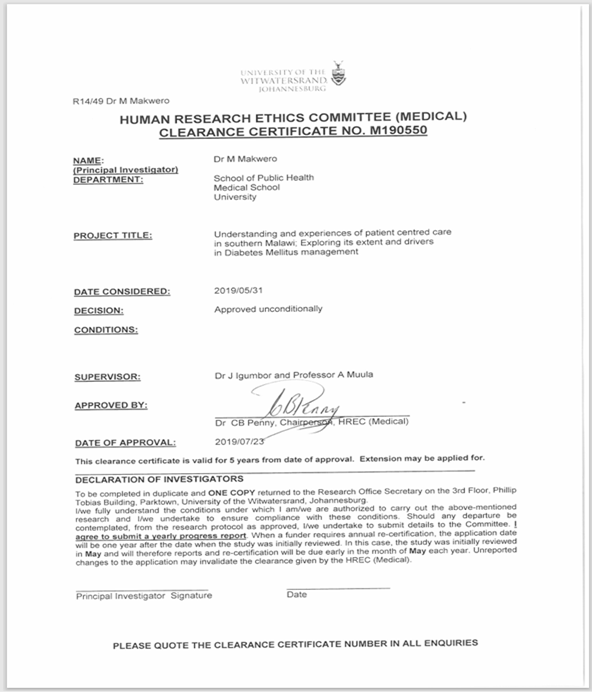


## Appendix 5: Information leaflet

**STUDY: STAKEHOLDERS’ UNDERSTANDING AND EXPERIENCES OF PATIENT CENTRED CARE: A CASE STUDY OF DIABETES MELLITUS MANAGEMENT IN SOUTHERN MALAWI.**

Dear Sir/Madam,

I, Dr Makwero and my research team are doing a study that aims to explore what patient centred care for diabetic patients is through understanding the perceptions and experiences of patients, providers and policy-makers and to assess the its practice and outcomes of patient centred care for diabetic patients in Malawi. This study will be done in Zomba, Chikhwawa, Mulanje and Blantyre districts diabetic clinics. The results will help us find out what diabetic’s patients actually expect from the health care system in order to inform quality improvement efforts in Malawi

**Invitation to Participate and What is involved in the study?** I invite you to participate to a focus group discussion to describe your perceptions and experiences of the care you receive at this facility. It is a study which will be incorporated into your routine care. On your regular appointment date, you will be asked to describe your experiences, expectation of care through an interview with myself. The interviews will be audio-recorded. Your visit may be one hour longer than usual.

**Risks and benefits:** There are no anticipated risks in the study since you will not be subjected to any strange drugs and tests. It will be beneficial that you to participate since your opinions will provide contributions to the understanding of what patient centred care is at this facility. You will be informed of the study results if you so wish.

**Participation is voluntary** and refusals to participate will not result in penalties or compromise to your entitled care for the day. You are free to withdraw from the study at any time without negative effects in the care rendered to you.

**Reimbursement**: As a token of appreciation, your transport today will be reimbursed using the Malawi standard rate of 3000 Malawi kwacha.

**Confidentiality:** Efforts will be made by the researcher to keep your personal information confidential unless if required by the law or governing bodies such as ethics committee for medical research or quality assurance purposes. However, confidentiality may not be fully guaranteed as information may be leaked by other members of the team.  In case the results of this study are published, that is when it may lead to cohort identification.

**Ethical approval:**  Permission to conduct the study and ethical approval has been obtained from COMREC and respective hospital boards. The respective letters are available if you need to have a copy.

**Contact details:** Should you have any questions or clarifications about the study, please contact the researcher or the COMREC Secretariat using the contact details written below:

Dr Martha Makwero                     The Research and Ethics Committee

College of Medicine (COM), Private Bag 360, Malawi COM Research Ethics committee

+265 1871911 (O)                                                       University of Malawi, P/Bag 360

+265 884111312(C)                                                                     Blantyre   Tel: +265 1871911

**Appendix 6:  Informed Consent form for interviews and audio-recording**

Informed consent form for patients, HCWs and policy makers

***(****To be translated into Chichewa)*

You have been asked to participate in this research study interview and audio-recording of the same).

You have been informed about the study by Dr M. Makwero (*or name of research assistant*)

If you have questions about the way the study is being conducted or your rights as a research subject you may contact any of the following

- Dr M Makwero at College of Medicine on telephone number 01871911 or cell phone 0884111312 at any time
- The Secretariat of the Ethics Committee of the college of Medicine ( COMREC), UNIMA on 01871911
- The chairperson of the human research ethics committee (HREC), Witwatersrand University; Professor Clement Penny on +27 11 717 2301, or by e-mail on [Clement.Penny@wits.ac.za](mailto:Clement.Penny@wits.ac.za). Alternatively, the committee secretariat on +2711 717 2700/1234 and the e-mail addresses are [Zanele.Ndlovu@wits.ac.za](mailto:Zanele.Ndlovu@wits.ac.za) and [Rhulani.Mukansi@wits.ac.za](mailto:Rhulani.Mukansi@wits.ac.za)

Your participation in this study is voluntary, and you will not be penalized or lose benefits if you refuse to participate or decide to terminate participation.

If you agree to participate, you will be given a signed copy of this document as well as the participant information sheet, which is a written summary of the research.

*The research study, including the above information, has been verbally described to me. I understand what my involvement in the study means and I voluntarily agree to participate in the* ***interview****.*

*……………………………………. ……………………………..*

*Signature of participant Date*

Signature of researcher (or assistant) administering the patient information.

Signature of Research assistant Date

**…………………………………………… …………………………**
