# Supplementary material for: Perception of patient-centred care and its relationship with management outcomes and complications among patients with DM in Malawi
Source: BMJ Open. 2025 Jul 5;15(7):e090308. doi: 10.1136/bmjopen-2024-090308 (PMC12228445; doi:10.1136/bmjopen-2024-090308)
Supplement: Supplementary file 2 [file bmjopen-2024-090308.reviewer_comments.pdf]

PEER REVIEW HISTORY

BMJ Open publishes all reviews undertaken for accepted manuscripts. Reviewers are asked to complete a checklist review form and are provided with free text boxes to elaborate on their assessment. These free text comments are reproduced below.

ARTICLE DETAILS

Title (Provisional)

The perception of patient-centred care and its relationship with management outcomes and complications among patients with Diabetes Mellitus in Malawi

Authors

Makwero, Martha Thokozani; Muula, Adamson S.; Anyanwu, Felix Chima; Maposa, Innocent; Igumbor, Jude

---

VERSION 1 - REVIEW

**Reviewer**	**1**
**Name**	**Nguyen, Anna**
**Affiliation**	**The University of Oklahoma Health Sciences Center, Fran & Earl Ziegler College of Nursing**
**Date**	**24-Sep-2024**
**COI**	**None**

---

1. The abstract mentioned adherence as an outcome measure but did not mention glycemic control - this was discussed in the paper as an outcome measurement.

2. There seems to be an incomplete sentence starting with line #49.

3. While the authors discussed reliability of the data collection tools (PCC, Self-efficacy, Adherence), there was no discussion on the tool validity.

4. The results on Adherence seem to be incomplete. Adherence was defined by the authors to include medication, diet, lifestyle, and appointment keeping. There was no data and no discussion on each of these components.

5. There were many abbreviations used without spelling them out initially.

6. Another limitation the authors should address is the self-report data - Potential for bias due to social desirability or respondent burden.

7. It is unclear how and who administered the questionnaires.

8. The authors used the term dependent/independent variables and the term endogenous/exogenous variables interchangeably. Use one or the other and be consistent.

Reviewer 2

Name Sheng, Chang-Sheng

Affiliation Shanghai National Clinical Research Center for Endocrine and Metabolic Diseases, Ruijin Hospital, Shanghai Jiaotong University School of Medicine, Shanghai, China

Date 25-Sep-2024

COI None

This comprehensive study delved into the intricate relationship between the Patient-Centred Care (PCC) concept and diabetes management, with a particular focus on its impact on treatment adherence among diabetes patients. The research provided valuable insights into how PCC approaches can influence patient outcomes and overall care quality.

Specifically, we would have appreciated a more in-depth examination of various diabetes medication treatments and their potential interactions with PCC approaches. An analysis of how different drug regimens might influence patient adherence within a PCC framework could have provided additional valuable insights. Furthermore, investigating whether the effectiveness of PCC varies across different types of diabetes medications could have offered a more nuanced understanding of its applicability in diverse treatment scenarios.

VERSION 1 - AUTHOR RESPONSE

	Comments	Response	Line number
Reviewer 1			
Abstract<br><br>1	Failure to mention Glycaemic control as an outcome	Glycaemic control was mentioned as an outcome in the abstract (line 33-34) however adherence was the main outcome	24, 25 , 48
2	Incomplete sentence on line 49	corrected	
3	Validity of the PCC, self-efficacy, and adherence data collection tools	This has been highlighted and references provided	PCC - 180<br>Self-efficacy (185-6) Adherence (197)
4	No data on the individual components of the adherence tool	The tool was used as a whole. It was not disaggregated. It was beyond the scope of the study	201-2
5	Abbreviations not explained	Corrected	91, 93

6	Added limitations on social desirability	It is added	78
7	Not clear who administered the questionnaire	Added	172
8	The use of dependent/independent Vs exogenous/endogenous variables synonymously	Corrected to exogenous/endogenous	line 228-229
	Reviewer 2		
1	a more in-depth examination of various diabetes medication treatments and their potential interactions with PCC approaches	There is no statistically significant relationship between PCC and type of medication. This is why this relationship was not explored further<br>It was only self-efficacy that had a statistically significant relationship with the type of medication. It is acknowledged in the paper	Table 1 & 2<br>342 & 378
2	An analysis of how different drug regimens might influence patient adherence within a PCC framework could have provided additional valuable insights	There is a statement about adherence and treatment regimes	Page 26, Table 4
3	Furthermore, investigating whether the effectiveness of PCC varies across different types of diabetes medications could have offered a more nuanced	There is no statistically significant relationship between PCC and type of medication. This is why this relationship was not explored further	

VERSION 2 - REVIEW

Reviewer 2

Name Sheng, Chang-Sheng

Affiliation Shanghai National Clinical Research Center for Endocrine and Metabolic Diseases, Ruijin Hospital, Shanghai Jiaotong University School of Medicine, Shanghai, China

Date 14-Apr-2025

COI

The authors have adequately addressed all my concerns, and I have no additional comments.
